# Global Variation in Zooplankton Niche Divergence Across Ocean Basins

**DOI:** 10.1111/ele.70089

**Published:** 2025-02-20

**Authors:** Niall McGinty, Andrew Irwin

**Affiliations:** ^1^ Department of Oceanography Dalhousie University Halifax Canada; ^2^ Department of Mathematics & Statistics Dalhousie University Halifax Canada

**Keywords:** bathymetry, copepods, HGAM, niche conservatism, temperature

## Abstract

Modelling responses to climate change assumes zooplankton populations remain similar over time with little adaptation (niche conservatism). Oceanic barriers, genetic, phenotypic variation and species interactions in cosmopolitan species could drive niche divergence within species. We assess niche divergence among 223 globally distributed species across the seven main ocean basins. There were 357 diverged niches out of 828 ocean basin comparisons. The proportion of diverged niches varied both across and within phyla. *Copepoda* (156 of 223 species) were used to test for niche divergence between same‐species populations across different environmental gradients. Global niche divergence was found to be more likely for species in colder temperatures and nearshore environments. Opposing temperature responses were found for four comparisons, which may relate to the different connectivity patterns between them. This study demonstrates adaptive potential across environmental‐niche gradients, which must be considered when modelling population responses to climate change.

## Graphical abstract

We explored the evidence of niche divergence between population for marine zooplankton globally. We found 44% of all population comparisons show niche divergence. Niche divergence varied across temperature and bathymetry gradients for copepods.

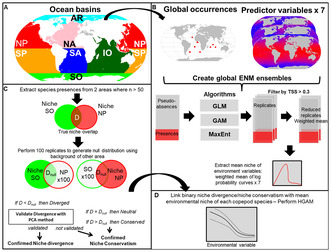

## 1 Introduction

Niche conservatism is the tendency for closely related species and clades to retain their ecological niche and traits over time (Wiens et al. 2010). A species' ecological niche is expected to change slowly so that species retain their ancestral traits (Wiens and Graham 2005; Wiens et al. 2010) remaining constant over thousands of years across a range of environments (Peterson 2011). The concept of niche conservatism has received attention recently due to its potential role in a wide range of ecological questions, including climate change responses (Pearman et al. 2008; Wiens et al. 2010), invasive species (Petitpierre et al. 2012) and causes of the global latitudinal diversity gradient (Romdal et al. 2013). In contrast, niches of closely related taxa may be more dissimilar than expected by chance and have therefore diverged (Hua and Wiens 2010). Evidence supporting niche divergence or conservatism appears to be mixed; however, evidence of either hypothesis appears to be a consequence of the resolution, both spatial and temporal, that is used in the analysis (Hu et al. 2015). Areas that have a greater heterogeneity in climate and habitat have a greater potential to promote niche divergence (da Silva et al. 2020). Here, we evaluate the likelihood of niche divergence for hundreds of zooplankton taxa by comparing their biogeographical distribution across ocean basins.

It is important to first consider what the ‘niche’ is in this context due to the broad range of definitions in use (Soberón and Peterson 2020). The ‘Grinellian’ niche defines the non‐interacting environmental conditions at broader scales that are more readily available for niche modelling (Soberón 2007). In contrast, the ‘Eltonian’ niche is a resource‐based interpretation of the niche where biotic interactions influence the niche but are more difficult to define (Soberón 2007). The advent of satellite measurements allows for resource‐based proxies to be incorporated (e.g., chlorophyll‐*a*) along with environmental or abiotic variables at similar scales. Combining both variable types allows us to view the realised ‘niche’ sensu Hutchinson (1957) which is an *n*‐dimensional hypervolume that defines the range of environmental conditions and biotic interactions that allow a species to grow, reproduce, and persist. We use here the Hutchinson definition of the realised ‘niche’ as it provides more information on the modes of niche conservatism (McCormack et al. 2010) and facilitates the understanding of niche conservatism (divergence) across environmental gradients (Colwell and Rangel 2009).

While evidence for or against niche conservatism has focused on terrestrial organisms, much less is known for pelagic organisms including zooplankton. The long‐held opinion has been that due to their large population sizes and means for high dispersal, the genetic structure between populations remains relatively mixed (Norris 2000). While early studies appeared to reinforce this finding, they were often concentrated on populations separated by short open‐ocean distances (e.g., Jarman et al. 2002). Subsequent studies on the population genetic structure have begun to explore the genetic differentiation of species with circumglobal distribution. These studies show that there are limitations to gene flow with genetic differentiation observed between populations across a broad range of zooplankton groups including Chaetognatha (Peijnenburg et al. 2006), Cnidaria (Dawson and Jacobs 2001), Pteropoda (Burridge et al. 2019) and Copepoda (Goetze 2003). Although significant genetic variation can occur across shorter distances, particularly in the presence of extreme environmental gradients (González et al. 2020), populations appear more stable within an ocean basin (Provan et al. 2009) with significant variation observed between basins (Goetze and Ohman 2010; Hirai et al. 2015). A strong genetic barrier appears to separate the North and South Atlantic at the equator (Hirai et al. 2015; Goetze et al. 2017) while another appears to occur between the Indian Ocean and Pacific Ocean (Goetze 2011). The findings suggest that large population numbers and short generation times result in relatively high adaptive potential of marine zooplankton which could manifest on ecologically meaningful timescales (Peijnenburg and Goetze 2013). Significant genetic variation between populations does not always equate to niche divergence. Though genetically distinct populations of *Pleuromamma xiphias* were found to have different thermal ranges (Goetze 2011), the pteropod *Cuvierina* sp. showed that genetic differentiation does not always imply niche divergence (Burridge et al. 2015). It appears niche conservatism is likely driven by several interacting forces that include both genetic variation and competitive interactions within the community (Chivers et al. 2017; McGinty et al. 2021).

Niche conservatism has important implications for how zooplankton will respond to the changing climate. Marine zooplankton have been identified as sentinels of climate change due to their short generation times and, as ectotherms, much of their vital life history traits are tightly coupled to changes in ocean temperature (Dam 2013). As a result, zooplankton have responded rapidly to changes in ocean temperature by shifting their distribution to track their thermal niche. Zooplankton have been shown to shift their distribution in response to increasing temperatures by shifting polewards (Beaugrand et al. 2002; Villarino et al. 2015). Localised responses of zooplankton to increasing temperatures have led to shifts in the phenology and timing of their life history (Schlueter et al. 2010). The ability for zooplankton species to actively track their ideal thermal niche rests on the tendency of zooplankton species to maintain niche conservatism with little adaptation (Beaugrand et al. 2014; Benedetti et al. 2021). Moreover, a species is also assumed to respond similarly to changing environmental conditions across its natural distributional range (Smith et al. 2019). The evidence that zooplankton are capable of rapid adaptive and evolutionary responses means that a species might consist of cryptic species complexes or locally adapted populations with varied responses (Peijnenburg and Goetze 2013). Given the fact that much of our understanding of zooplankton dynamics relies on the vast historical databases of morphologically identified species that could miss the local adaptation of populations within some species (Blanco‐Bercial et al. 2011), it is important that we understand what factors might facilitate or constrain niche divergence and the relevant spatial and temporal scales (Choo et al. 2023).

## 2 Materials and Methods

**FIGURE 1:**
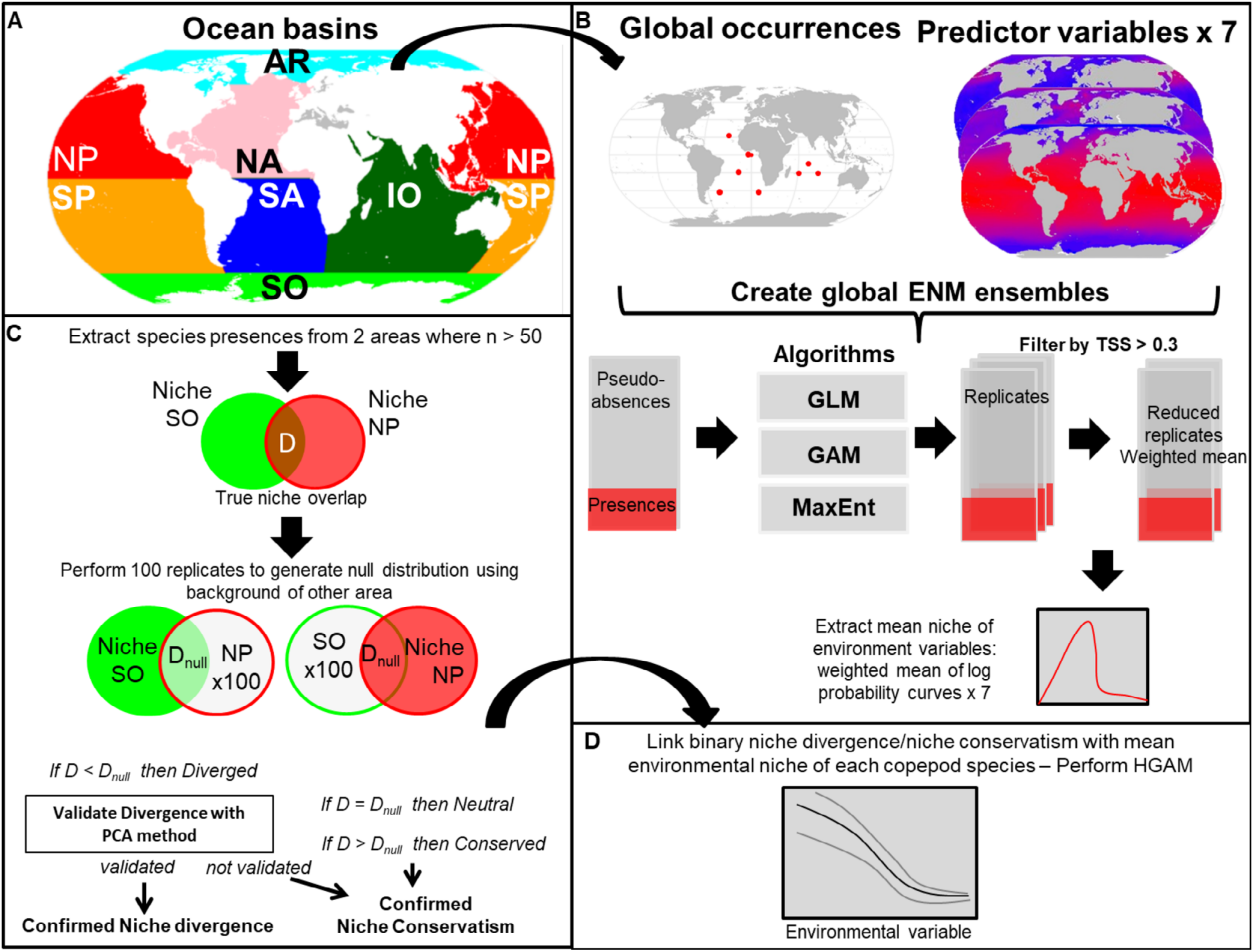
The workflow outlining the process for estimating niche divergence of species occurrences in different ocean basins. (A) The delineation of the seven ocean boundaries used for the paired area comparisons (Arctic Ocean = AR, Indian Ocean = IO, North Atlantic = NA, North Pacific = NP, South Atlantic = SA, South Pacific = SP, Southern Ocean = SO). (B) Construct global ENM ensemble for all species: Step 2—Calculate niche overlap (D) of two populations of the same species in two paired areas. (C) Estimating niche overlap and background similarity between paired area observations by generating 100 null model replicates. Evidence of niche divergence is validated by a principal component analysis if niche divergence is evident. (D) Paired area comparisons for Copepoda are assessed for potential signals in niche divergence using a hierarchical GAM.

For brevity, we present an overview of the main methodological processes in this section but refer the reader to (Note S1) which provides expanded detail and information on the workflow. Figure 1 summarises the processes and framework used in constructing the niche divergence analysis across ocean basins. All analyses were performed using the R programming environment, and the packages used are referenced in the appropriate sections.

### 2.1 Zooplankton Data

Zooplankton occurrence data were sourced from the “Zoobase” database (Benedetti et al. 2021), which compiles observations from different repositories for main zooplankton taxonomic groups that comprise the majority of meso‐zooplankton biomass globally. To reduce potential sampling bias, data underwent spatial filtering according to Benedetti et al. (2023), limiting occurrences to those collected from the upper 200 m of the ocean. Data were then thinned to retain a single observation per 0.5° grid cell per month, aiming to reduce redundancy in highly sampled areas. Species with at least 50 observations in a minimum of two ocean basins were included in the niche divergence analysis. This resulted in 267 species with sufficient occurrences, yielding 789,231 unique observations for subsequent modelling (Figure S1).

### 2.2 Environmental Variables Selection

The selection of environmental predictors involved identifying biotic and abiotic variables with demonstrated influence on zooplankton distribution, whether by direct effects on metabolism (e.g., temperature) or indirect proxies for food availability (e.g., chlorophyll‐a). Candidate variables included temperature (°C), salinity, nitrate, dissolved oxygen concentration, mixed layer depth (MLD), wind stress, chlorophyll‐a, and bathymetric depth (Table S1). These variables were extracted using the R packages raster and ncdf4 and integrated into monthly climatologies on a 1° grid resolution. Temperature, salinity, nitrates, and dissolved oxygen concentration were averaged across the upper 200 m, while monthly wind stress data with 0.25° resolution were obtained from the Aviso dataset, representing sub‐mesoscale dynamics and eddy formation. Chlorophyll‐a data, serving as a primary productivity proxy, came from the GlobColour database, with data averaged to match the 1° resolution. Bathymetric depth was derived from the GEBCO topographical dataset.

Environmental predictor choices were further assessed for sampling effort imbalance and potential multicollinearity. The greatest imbalance was noted in salinity due to the low‐salinity environments in areas like the Baltic and Black Seas. Multicollinearity was evaluated via Spearman correlation and variance inflation factor (VIF), using thresholds of *r* < 0.75 and VIF < 5, respectively (Dormann et al. 2013). Dissolved oxygen concentration was removed due to its high correlation with temperature (*r* = 0.91, VIF = 17.5). The retained variables included temperature, salinity, nitrate, MLD, chlorophyll‐a, wind stress, and bathymetry. Zooplankton data were linked to environmental data based on matching monthly climatologies within a 1° spatial grid.

### 2.3 Study Area Selection

As a priori information on genetic population structure is limited for most species, we look to define populations by minimising the mean geographic distance between species observations within areas, in contrast with the mean global mean geographic distance between observations. We used ocean basins to define areas, as the mean geographic distance within ocean basins was consistently less than those from data‐defined clusters (Note S1; Figure S2). We limited our observations to the seven main ocean basins: North Atlantic, South Atlantic, North Pacific, South Pacific, Indian, Arctic, and Southern Oceans. We removed the marginal seas with unique salinity profiles (i.e., –Mediterranean, Baltic and Black Sea) to offset the sampling imbalances in environmental space of salinity. We also applied a buffer of 0.5° at the boundaries of ocean basins to remove any potential biases from shifting oceanographic boundaries between basins (e.g., the Antarctic circumpolar current). Our final dataset consisted of 223 species from 642,364 observations (Table S2).

### 2.4 Environmental Niche Model Ensembles (ENM)

We use the ODMAP protocol (Overview, Data, Model, Assessment and Prediction; Zurell et al. 2020) to maximise reproducibility and transparency when developing ENM, details of which can be found in (Table S3). Each species' global ENM was built on the selected environmental variables, ensuring at least 100 observations per species, which aligns with the recommended minimum ratio of observations to predictor variables (10:1—Guisan et al. 2017). The ENMs were created using the R package biomod2, which integrates three modelling algorithms to generate an ensemble model for each species: Maximum entropy (MaxEnt), generalised linear models (GLM), and generalised additive models (GAM). These algorithms capture a range of species‐environment relationships, from linear to machine‐learning methods, and are widely used for niche overlap studies. MaxEnt was limited to default settings, with a regularisation multiplier of 1 and linear/quadratic transformations only (Valavi et al. 2023). GLMs used a logistic link function without variable interactions, while GAMs applied a smoothing function with a four degrees‐of‐freedom limit to prevent overfitting.

Randomised background point selection was modified to avoid model bias (Lobo et al. 2010). Background locations were selected using a targeted group approach in proportion to zooplankton occurrence density, maintaining a 10:1 background to presence ratio (Phillips et al. 2006). For species with many presences, background samples were capped at 1000 points (Barbet‐Massin et al. 2012). Model validation was performed via block resampling, dividing the dataset into spatially structured training and testing blocks using the blockCV package. Evaluation metrics included the true skill statistic (TSS) and the continuous Boyce index (CBI), which are better suited for presence‐only data (Allouche et al. 2006; Hirzel et al. 2006). TSS combines sensitivity and specificity, with values below zero indicating no better performance than random. CBI, ranging from −1 to 1, assesses correlation between predicted and observed presence probabilities, with values > 0 indicating a positive correlation. Only models with a TSS greater than 0.3 were retained in the final ensemble model, thereby removing poorly fitted models based on the block resampling validation. The models were weighted using a weighted average procedure, which averages predictions based on the performance of individual models (i.e., strongly performing models influence the ensemble average the most). The resulting ENMs allowed us to extract mean niche values for each species, using the logistic probability of a species presence as the weighting factor along each environmental gradient. These mean niche values are combined with the niche divergence assessments for further modelling (see below).

### 2.5 Niche Divergence Assessment Using ENM

To test for niche divergence, we used a two‐part analysis combining ENM and principal component analysis (PCA) following McCormack et al. (2010) and McGinty et al. (2021). Step 1 involved comparing ENM ensembles for species in paired ocean basins using Schoener's D metric, where 0 represents no overlap and 1 indicates perfect overlap. We used the ENMtools package to perform a background similarity test to detect if niche differences were due to background environmental variation. Ocean basin pairs were given four‐character identifiers (e.g., North Atlantic and North Pacific—NA‐NP). To evaluate divergence, a null model was generated by comparing presence data from one ocean basin against background data from the other, repeated 100 times to create a null D distribution (D_null_). True niche overlap values of D falling outside the 95% CI of D_null_ were interpreted as evidence of niche divergence (D<D_null_) or conservatism (D>D_null_).

### 2.6 Niche Divergence Assessment Using PCA

PCA provided additional validation of niche differences and insight into the environmental variables driving niche shifts. For species with over 1000 observations in a basin, a subset of 1000 was selected to construct the PCA. Environmental data from both basins were matched, maintaining a 10:1 background‐to‐species presence ratio. An elbow plot was used to determine the principal components explaining the majority of variation. Niche overlap was measured by the difference in mean PCA scores for each ocean basin. Null distributions for each axis were created using 1000 jack‐knife replicates, with niche divergence confirmed if mean differences exceeded the 95% CI of null values. A Bonferroni‐corrected *t*‐test verified significance across all species and area combinations.

### 2.7 Addressing Sampling Bias and Variation

The null model framework also facilitates an unbiased assessment of niche overlap in the presence of imperfect sampling of the environmental space in both areas. Null models generate the ‘expected’ distribution based on the known environmental space in both areas. By comparing niche overlap to the expected random distribution, you can determine if species are selecting environments non‐randomly given the expected differences in environmental variability (Warren et al. 2008). Further sampling biases, potentially arising from imbalanced species observations across basins, were tested by resampling presence points for each population, adjusting observation numbers from 25 up to 1000. Niche overlap (D) was calculated for each resampling level, allowing us to assess if divergence stemmed from sampling variation. A logistic regression was also conducted, comparing divergence versus non‐divergence with observation differences between basins, to examine if observation frequency influenced niche classification.

### 2.8 Environmental Drivers of Niche Divergence in Copepods

Copepod comparisons were available in suitable numbers to examine environmental influences on niche divergence between basin pairs. We combined the binary niche divergence classifications of each paired area comparison with mean niche values from global ENMs, which describe each species' preferred environmental gradient. Hierarchical generalised additive models (HGAM) tested whether niche divergence likelihood was related to environmental affinities. HGAMs extend traditional GAMs by accounting for group‐level predictor‐response relationships (Pedersen et al. 2019). Model selection used a penalty‐based approach, simplifying the final model by removing insignificant smoothers. With limited data between paired areas, estimates of both fixed and random effects may become biased, and as a result, we excluded paired areas with < 10 comparisons for copepods, resulting in 14 of the 21 areas retained and 552 paired area comparisons. After selection, the final model was of the form:

$$\begin{array}{c} \text{Div}\sim \text{s}\left(\text{Bathymetry,}\;k=4\text{,}\;\text{bs}=\;“\text{tp}”\right)\\ +\text{s}\left(\text{Temperature},\;\text{kn}=4,\;m=2,\text{bs}=“\text{tp}”\right)\\ +\text{s}\left(\text{Temperature},\;\text{by}=\text{Paired Areas},\;\text{kn}=4,\;m=1,\;\text{bs}=“\text{fs}”\right)\\ +\text{s}\left(\text{Paired Areas,}\;\text{bs}=“\text{re}”,k=12\right) \end{array}$$

The model fits a global level trend for the bathymetry and temperature. Temperature also allows for different group‐level trends with different smoothness between different paired areas. Paired areas will show significance with temperature if the group‐level trend is significantly different from the global pattern. We also included the paired areas as a random effect to account for the variation of species between paired areas.

## 3 Results

**FIGURE 2:**
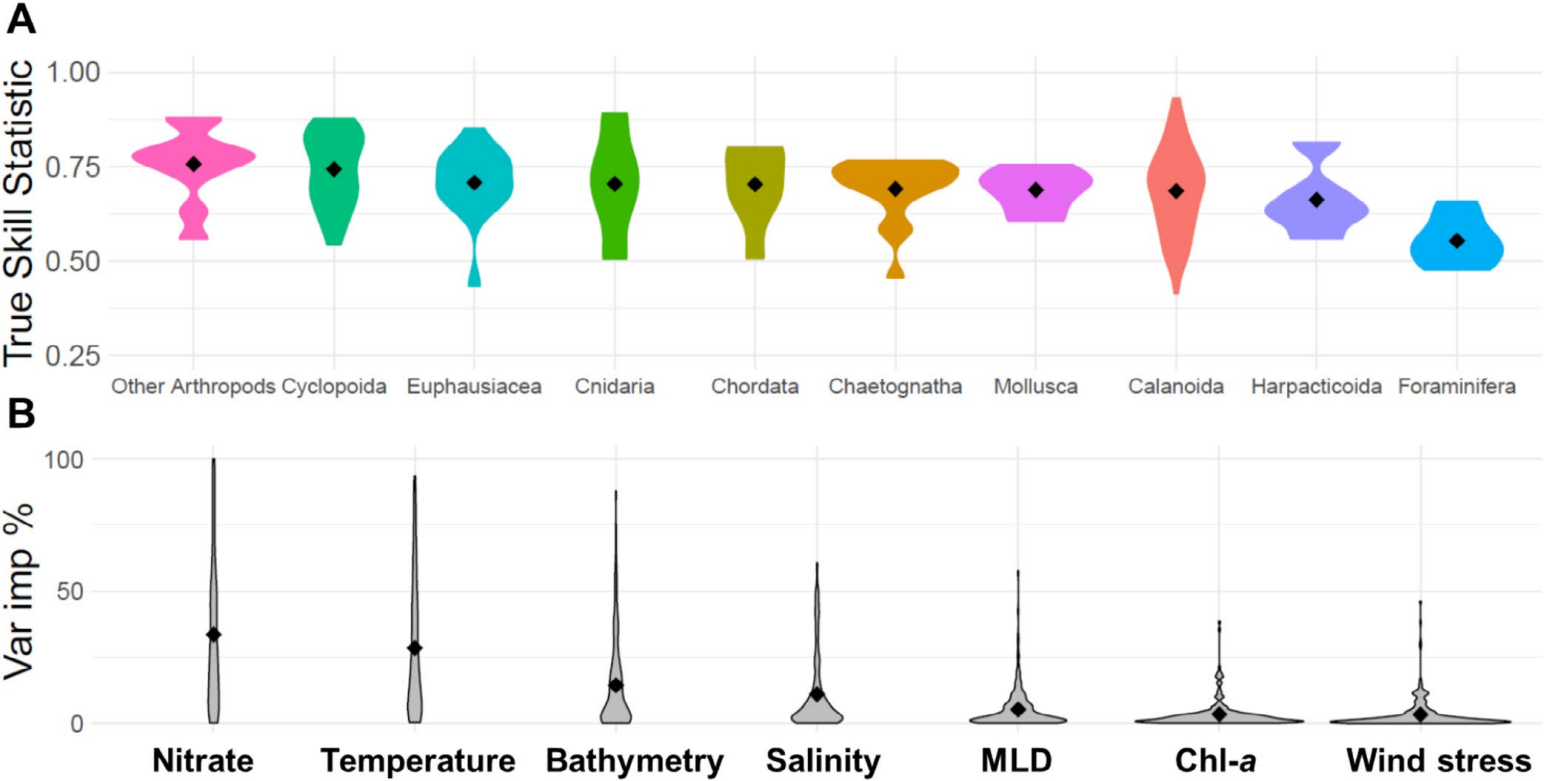
A ribbon plot showing the mean (points) and range of the (A) true skill statistic (TSS) and for 10 of the main taxonomic groups. A ribbon plot showing the permutation importance (B) of the seven variables used in constructing the global ensemble models, ordered from most important to least.

The performance of the three algorithms used for the ensemble model was largely consistent across the different taxonomic groups (Figure S3). The ensemble model performance varied across the 10 different taxonomic groups that were defined (Figure 2). The highest TSS was found for the copepod group Cyclopoida and other Arthropods with mean values of 0.74 ± 0.07 and 0.75 ± 0.11 respectively. In contrast, the lowest TSS values were found for Foraminifera with a mean value of 0.56 ± 0.07 (Figure 2A). There was a large degree of variability across species regarding the variable importance of each of the seven variables used in constructing the global models (Figure S4). However, temperature and nitrate were the most important predictors in almost half of all 223 models, with a mean importance of 27.2% and 33%, respectively (Figure 2B).

Of the remaining variables, only bathymetry has a mean importance greater than 14% and is the most important variable in more than 32 of the 223 global ensemble models. The background environmental conditions within each area had a strong degree of overlap except for temperature and nitrate (Figure S5). The regions SO and AR had a much lower temperature ranges in contrast with other basins while SO had significantly higher nitrate concentrations compared with other basins.

**FIGURE 3:**
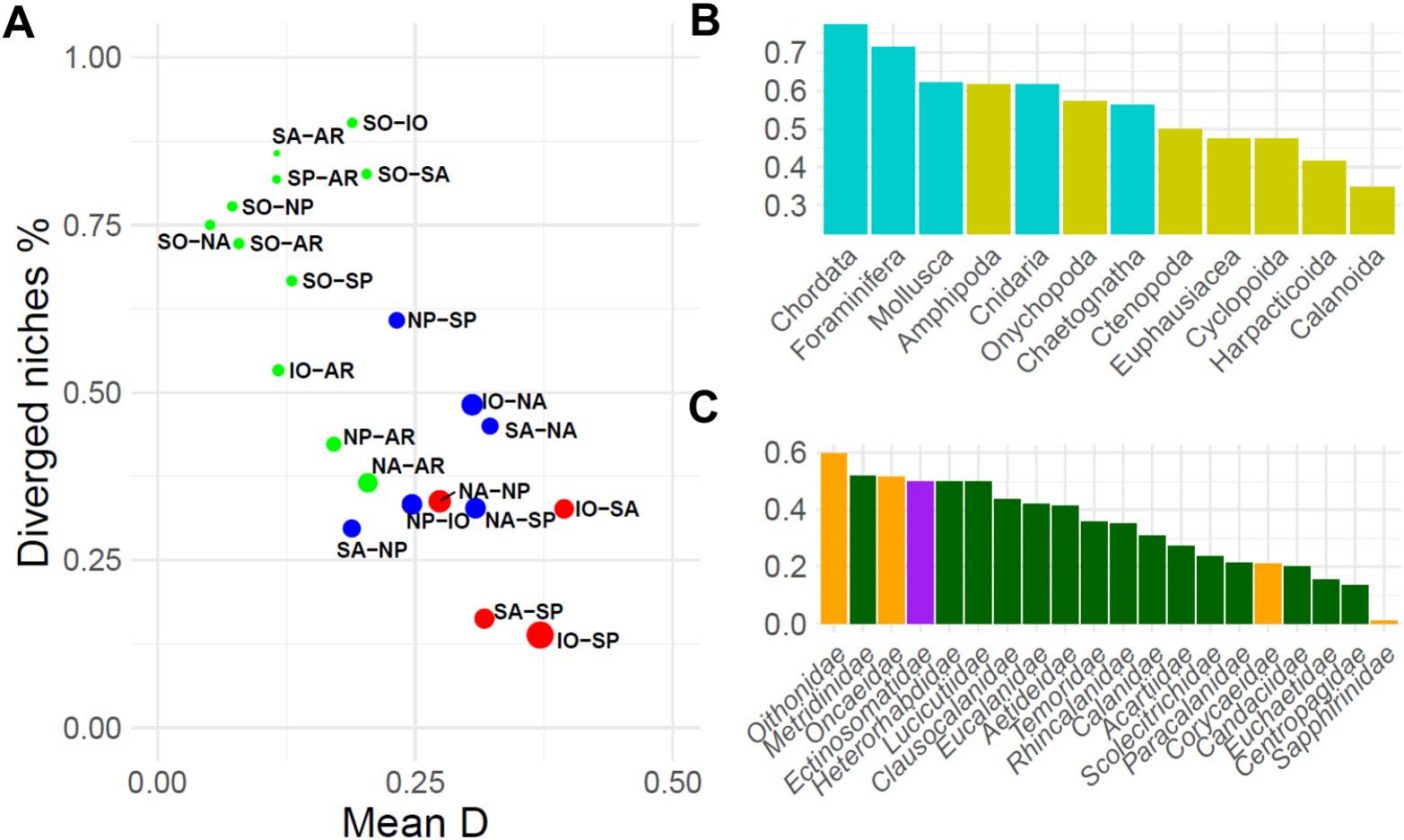
(A) The mean Schoener's D (Mean D) and the percentage of diverged niches (Diverged niches %) for each of the 21 paired area comparisons. Points are sized based on the total paired area comparisons made. The points are coloured based on whether the paired area comparisons were performed between areas in the same hemisphere (red), different hemispheres (blue) or between the Arctic/Southern Ocean and other areas (light green). (B) The percentage of diverged niched for the taxonomic groups. Bars are coloured according to the taxonomic resolution of each group with Phylum—cyan and Arthropod Class—yellow. (C) The percentage of diverged niches for copepod families across all paired area comparisons. Bars are coloured according to Order with Cyclopoida—orange, Harpacticoida—purple and Calanoida—dark green.

A total of 828 comparisons were made across the 21 paired areas, with total comparisons varying between 94 (IO‐SP) and 7 (SA‐AR). There were 357 diverged niches (43%), with the proportion ranging from 13% between IO‐SP and 90% for SO‐IO. Of the remaining comparisons, only 25 (3%) were found to be fully neutral. As a result, conserved and neutral niches were combined to create a binary variable of diverged and non‐diverged niches. The percentage of diverged niches was negatively related to the mean niche overlap D of species across the paired areas (Figure 3A). The PCA was broadly similar across the different paired areas. The elbow plot identified that the first three principal components explain, on average, 72% of the variation across paired areas. The variance explained remained consistent across all paired areas for PC1 (32%), PC2 (21%) and PC3 (14%). For PC1, temperature was the most important variable for 8 of the 21 areas, with a mean correlation of *r* > ± 0.76. PC2 had weaker correlations, but chl‐*a* was the most important for 10 of the 21 areas, with a mean correlation of *r* > ± 0.35. PC3 correlated very strongly with at least one variable in each paired area, with temperature (|*r*| > 0.83) the most important in 12 of the 21 areas, while nitrate (|*r*| > 0.78) and chl‐*a* (|*r*| > 0.79) were most important for the remaining paired areas.

The number of paired area comparisons within each phylum varied considerably from Annelida (1) to Arthropoda (694) of which 615 were within the class Copepoda. The Chordata had the highest rates of niche divergence at 77% (Figure 3B). Of the 223 species used in the analysis, 156 belonged to the class Copepoda. There were 43% of all paired areas diverged, with the copepod groups of Cyclopoida and Harpacticoida showing niche divergence close to 50% of all comparisons, while 35% of Calanoida showed niche divergence (Figure 3B). A total of 91 of the 156 species showed evidence of divergence between at least 1 of the 21 pairwise comparisons of the species populations. The cyclopoid species *Oithona similis* diverged in 16 of all 21 paired area comparisons, while the calanoid copepod *Metridia lucens* diverged in 9 of 16 paired area comparisons. Between copepod families, there was a contrast in the number of diverged populations across paired areas. Most diverged niches occur in the families from the order Cyclopoida (*Oncaeidae* and *Oithonidae*) with 52% and 60%, respectively (Figure 2C). In contrast, the 15 comparisons from *Centropagidae* showed divergence in 13% of all measured (Figure 3C).

Differences in niche overlap comparing the full dataset of observations and estimates from a fixed number of observations were largely consistent for sample sizes greater than 50 (Figure S6). Differences in niche overlap were found to be, on average, less than 0.07 between 50 and 1000 observations. The observed differences in niche overlap did not re‐define the paired‐area comparison from diverged to conserved or vice versa. Differences in observation number did not have a significant effect on the classification of niche divergence. *R* ^2^ = < 1%; *F* 1826 = 0.789; *p* = 0.375.

**FIGURE 4:**
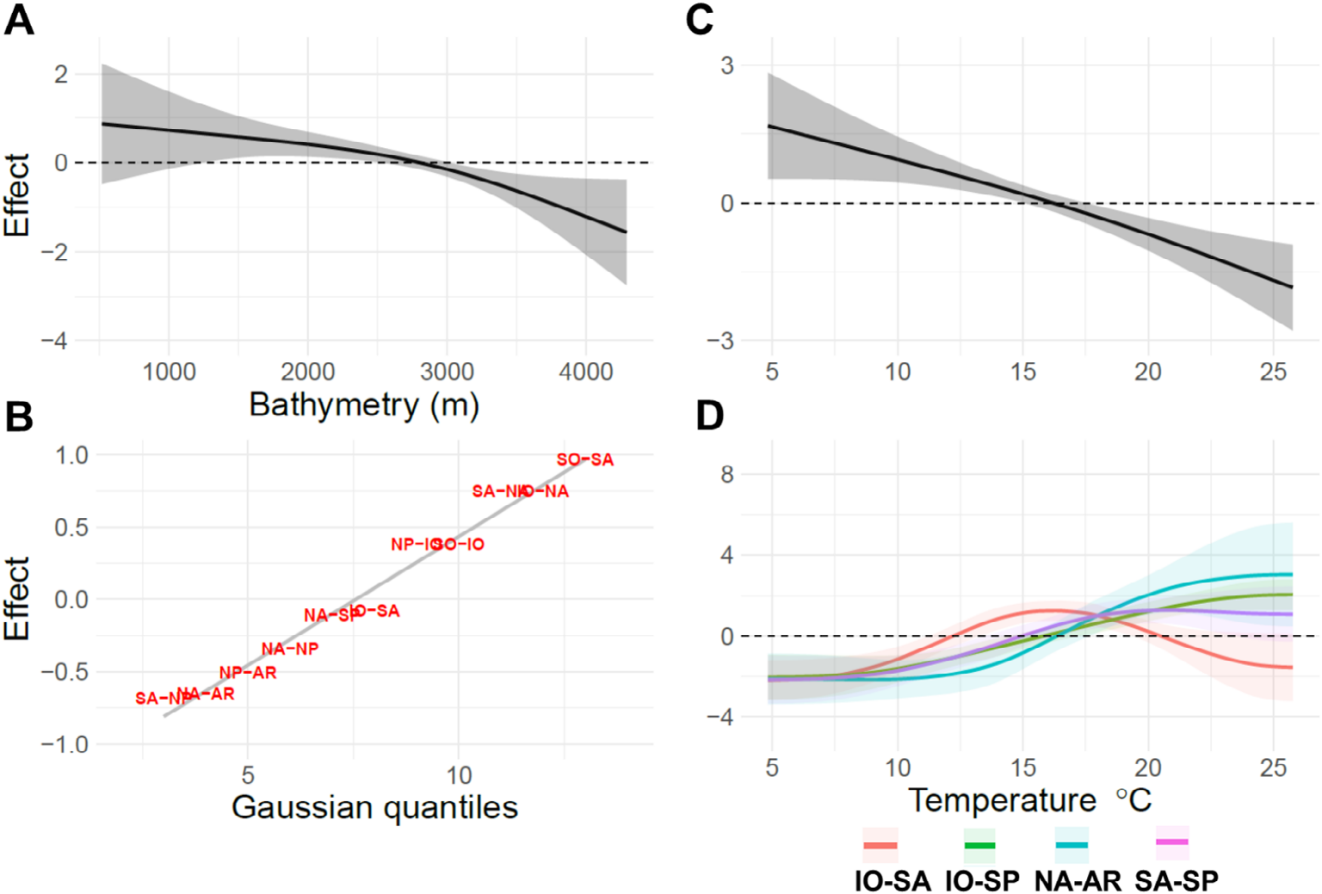
Hierarchical‐GAM smoothers that show the partial effect of niche divergence across significant environmental gradients. Values above 0 show niches that are more likely to represent species with diverged niches between two paired areas. (A) The global smoother of bathymetry, (B) the random effect size of paired area, (C) the global smoother for temperature and (D) significant paired area smoothers of ocean temperature that deviate significantly from the global smoother. The paired areas are IO‐NA—red, IO‐SA—gold, IO‐SP—green, NA‐AR—cyan, SA‐NP—pink and SA‐SP—purple.

We observed significant relationships between the probability of niche divergence of species populations and their mean temperature and bathymetric niches (HGAM; *R* ^2^ = 0.35; Table 1). A global smoother for bathymetry and temperature shows that populations that occur in cooler (~15°C and lower) and shallower waters are more likely to show evidence of niche divergence (Figure 4A,C). The random effect of the paired areas shows that the SO‐SA has a high number of diverged niches, while SA‐NA has the lowest compared to the effect average. Four paired areas displayed a trend significantly different from the global temperature pattern (Figure 4D). For IO‐SA, IO‐SP, NA‐AR, and SA‐SP, niches were more likely to be diverged at warmer temperatures in contrast to the colder temperatures of all other areas. SA‐SP shows an increased chance of diverged niches at intermediate temperatures between 10°C and 20°C before decreasing again at higher temperatures.

**TABLE 1:** The results of the HGAM showing significant smoother variables only. For each variable the estimated, reference degrees of freedom (Est. df and Ref. df), Chi squared (Chi sq) and probability (*p*) of each variable are shown. The total sample size (*n*) and adjusted *R* squared (Rsq adj) for the final model are also displayed.

Variable	Est. df	Ref. df	Chi sq	*p*
s(Bathymetry)	1.77	2.18	12.1	0.003
s(Temp)	1	1	20.37	< 0.001
s(Temp):IOSA	1.59	3	13.19	0.04
s(Temp):IOSP	2.41	3	33.93	< 0.001
s(Temp):NAAR	2.65	3	27.03	0.005
s(Temp):SASP	1.52	3	7.35	0.018
s(area pair)	1.02	13	21.95	< 0.001
Deviance expl.	33.20%		*n*=	552

## 4 Discussion

We found niche divergence in zooplankton populations across a broad range of phyla. Just under half of all pairwise comparisons of populations showed significant niche divergence. The proportion of populations with divergent niches varied across orders and families within each phylum (Goetze 2005). For copepods, we found that niche divergence was more likely to occur between populations in cooler and shallower waters (Figure 4). While these effects are significant, there is still a large source of variability left unexplained by our HGAM model. This uncertainty suggests that there are other drivers that promote niche divergence, which include ecological interactions between species (e.g., competition, predation, etc.), phenotypic adaptation within species (e.g., thermal limits, metabolism) and sampling variability (e.g., within basin variation).

Our results align with increasing evidence of cryptic speciation of several globally distributed zooplankton taxa, which show genetic divergence between populations that display adaptation to local environmental conditions (Blanco‐Bercial et al. 2014). The tropical Atlantic has been identified as a persistent barrier to zooplankton dispersal (Burridge et al. 2015, 2019; Goetze et al. 2017; Choo et al. 2021) while the equatorial subtropical boundaries in the Pacific also act as an effective barrier to copepods (Goetze 2005). Comparisons within the same hemisphere show a much lower likelihood of niche divergence and the highest niche overlap (Figure 3A). Similar patterns between the thermal niche and niche divergence of populations are found for NP‐NA that we find across the equator, while we find an opposite pattern between NA‐AR. The pattern suggests we should see an opposite pattern between temperature and niche divergence for the NP‐AR. While there is a large inflow and outflow of currents between the NA‐AR, currents between the NP‐AR are predominantly northward through the narrow Bering strait (Weydmann et al. 2016). This appears to limit gene flow between Pacific and Arctic/Atlantic populations, which is found with species such as *Calanus glacialis* (Nelson et al. 2009). In the southern hemisphere, we see increased niche divergence at warmer temperatures in contrast to the global pattern of increased niche divergence at colder temperatures for IO‐SP, IO‐SA, and SA‐SP (Figure 4D). The contrasting pattern is likely due to the increased connectivity at the southern reaches of these three ocean basins, where there are weaker ocean current barriers or continental landmass barriers. Weak genetic divergence was found in populations of the copepod *Haloptilus longicornis* between IO‐SA (Norton and Goetze 2013) and for *Eucalanus* spp. (Goetze 2005) and *Pleuromamma xiphias* (Goetze 2011) between the IO‐SP. Comparisons between the Southern Ocean and other paired areas showed the greatest number of diverged niches and the lowest mean niche overlap between species (Figure 3A). Strong circumpolar currents limit the dispersal of plankton across its boundary, defining the latitudinal boundary of many Antarctic species (Murphy et al. 2021) including genetic isolation as in the case of the copepod *Metridia lucens* (Stupnikova et al. 2013). The high degree of endemism in the Southern Ocean is highlighted by the limited species available to compare for niche divergence. Species across different taxonomic groups show distinct genetic adaptation to the unique cold‐water environment and sea‐ice dynamics (Johnston et al. 2022).

The niche divergence relationships between ocean basins highlight the mosaic of frontal and landmass barriers to dispersal and the strength of connectivity between them. The connectivity of the global surface ocean is on average less than a decade (Jönsson and Watson 2016). Metagenomic data for a wide range of prokaryotic and eukaryotic planktonic groups show that genetic similarity decreases with increased surface ocean travel time, with a distinct genetic structure for the Southern Ocean (Laso‐Jadart et al. 2023). Size appears to play a role in the strength of genetic similarity, where larger‐sized zooplankton tend to be structured by ocean basins and current boundaries, while smaller organisms respond to environmental heterogeneity (Sommeria‐Klein et al. 2021). Species turnover rates with respect to ocean travel time are also correlated with body size. For larger zooplankton, travel time and dispersal distances are much lower than smaller organisms, with distinct boundaries of turnover rates along ocean boundaries (Villarino et al. 2018). Our findings highlight a similar process in structuring niche divergence within species across ocean basins, where strong environmental gradients, in particular temperature, occur.

Ocean temperature is a primary driver in structuring the abundance and distributional patterns of zooplankton in the ocean (Hays et al. 2005; Villarino et al. 2020). Marine species are thought to be particularly sensitive to temperature changes as they live close to their thermal limits (Pinsky et al. 2019). Observations of *Calanus helgolandicus* showed little evidence of thermal adaptation over the last 50 years (Hinder et al. 2014), though experimental studies on copepods have shown that rapid multi‐generational adaptations to warming can occur, with trade‐offs in fitness observed (Dam et al. 2021). Copepod thermal tolerances are positively linked to temperature, with trade‐offs between the phenotypic plasticity of a species’ thermal limits and thermal tolerance strength (Sasaki and Dam 2021). A comparison of thermal tolerances within species shows a similar trade‐off pattern with phenotypic plasticity for *Acartia tonsa* (Sasaki and Dam 2019). The patterns of within‐species variation in thermal tolerance and their plasticity response appear to be common across many marine species, highlighting the different sensitivities of a population to temperature changes (Sasaki et al. 2022).

Combined with temperature, we find that there is a general pattern of greater niche divergence for copepod species in coastal environments (Figure 4). They show a greater likelihood of niche divergence, exhibiting more plasticity in their niche preferences, and several species have shown significant adaptive differentiation to temperature over small spatial ranges (Sasaki and Dam 2019). Niche divergence in cooler, shallower habitats can increase the vulnerability of these copepods to climate change. While indicating a historical adaptation to exploit specific niches through specific traits (e.g., thermal tolerances), it can increase the chance of genetic isolation of the species population, reducing the capacity for phenotypic plasticity (Sasaki and Dam 2021). As global temperatures rise, these species may face habitat loss if their preferred cooler temperatures are no longer available in shallow waters. Since these habitats are limited in spatial extent compared with the open ocean niches, they are already spatially constrained. Near‐shore copepods are likely under different ecological, dispersal, and evolutionary constraints than their open ocean counterparts, which often inhabit slower changing and stable environments, suggesting different thermal tolerances and adaptation between species in these habitats (McGinty et al. 2021).

Our study has shown significant niche divergence in a wide range of species, which should be considered when modelling changes in zooplankton biogeography. Lineage information, through pooling or splitting of species populations, may account for differences in niche conservatism across species (Smith et al. 2019). Overlooking the within‐species variability in niche divergence or plasticity will likely have a significant effect on the real changes to zooplankton populations in response to ocean warming (Sasaki et al. 2022). Niche divergence in copepods varies across thermal gradients, which will have important implications for how species populations respond to climate change at the limits of their ranges. The increased niche divergence for colder‐shallower species suggests a greater vulnerability of these groups to future climate change. While under niche conservatism, individuals of a species at higher latitudes would be expected to follow a similar thermal performance curve to lower‐latitudes individuals. We show that it is more likely that these are cold‐water specialists with a limited capacity to respond to warming (Sasaki and Dam 2021). Future studies could look to explore the mechanisms that drive niche divergence in the realised niches of these species. Empirical studies or fitness experiments could provide some further insight into the effects of niche divergence in the near future.

The genetic differentiation of species sub‐populations for copepods can occur within ocean basins and across short geographical distances where a sub‐population occupies a unique niche or habitat (González et al. 2020), Much of our information on the spatial distribution of genetic lineages are focused on very few taxa (e.g., *Pleuromamma*) with limited information for other species. As a result we might be (1) comparing separate populations, (2) combining sub‐populations or (3) comparing the same population between ocean basins. Without a full understanding of the population distinction regionally and within basins we choose to only focus on the differences between ocean basins. Given the near‐shore and off‐shore differences in niche divergence between ocean basins and the small‐scale variation in thermal adaptation for some species, we are likely underestimating the magnitude of niche divergence by smoothing over these differences. To understand niche variation at a more regional level, we need data on a wider range of zooplankton species to allow for a more nuanced delineation of ocean boundaries (e.g., ‘isolation by currents’—Richter et al. 2022) to quantify the importance of niche divergence at these scales.

## Author Contributions

N.M.G. and A.I. devised the study and the main conceptual ideas. N.M.G. developed the theory and performed the statistical analysis. Both N.M.G. and A.I. discussed the results and contributed to the final manuscript.

### Peer Review

The peer review history for this article is available at https://www.webofscience.com/api/gateway/wos/peer‐review/10.1111/ele.70089.

## Supporting information


Appendix S1.

Data S1.

Table S3.

Note S1.

## Data Availability

The data and code used in this manuscript have been made publicly available on Dryad at the link doi: 10.5061/dryad.nvx0k6f2v.

## References

[ele70089-bib-0002] Allouche, O. , A. Tsoar , and R. Kadmon . 2006. “Assessing the Accuracy of Species Distribution Models: Prevalence, Kappa and the True Skill Statistic (Tss).” Journal of Applied Ecology 43, no. 6: 1223–1232. 10.1111/j.1365-2664.2006.01214.x.

[ele70089-bib-0004] Barbet‐Massin, M. , F. Jiguet , C. H. Albert , and W. Thuiller . 2012. “Selecting Pseudo‐Absences for Species Distribution Models: How, Where and How Many?” Methods in Ecology and Evolution 3, no. 2: 327–338. 10.1111/j.2041-210X.2011.00172.x.

[ele70089-bib-0005] Beaugrand, G. , P. C. Reid , F. Ibanez , J. A. Lindley , and M. Edwards . 2002. “Reorganization of North Atlantic Marine Copepod Biodiversity and Climate.” Science 296, no. 5573: 1692–1694. 10.1126/science.1071329. 12040196

[ele70089-bib-0006] Beaugrand, G. , E. Goberville , C. Luczak , and R. R. Kirby . 2014. “Marine Biological Shifts and Climate.” Proceedings of the Royal Society B: Biological Sciences 281, no. 1783: 20133350. 10.1098/rspb.2013.3350. PMC3996605 24718760

[ele70089-bib-0007] Benedetti, F. , M. Vogt , U. Hofmann Elizondo , D. Righetti , N. E. Zimmermann , and N. Gruber . 2021. “Major Restructuring of Marine Plankton Assemblages Under Global Warming.” Nature Communications 12, no. 1: 5226. 10.1038/s41467-021-25385-x. PMC8410869 34471105

[ele70089-bib-0008] Benedetti, F. , J. Wydler , and M. Vogt . 2023. “Copepod Functional Traits and Groups Show Divergent Biogeographies in the Global Ocean.” Journal of Biogeography 50, no. 1: 8–22. 10.1111/jbi.14512.

[ele70089-bib-0009] Blanco‐Bercial, L. , J. Bradford‐Grieve , and A. Bucklin . 2011. “Molecular Phylogeny of the Calanoida (Crustacea: Copepoda).” Molecular Phylogenetics and Evolution 59, no. 1: 103–113. 10.1016/j.ympev.2011.01.008. 21281724

[ele70089-bib-0010] Blanco‐Bercial, L. , A. Cornils , N. Copley , and A. Bucklin . 2014. “DNA Barcoding of Marine Copepods: Assessment of Analytical Approaches to Species Identification.” PLoS Currents 6: ecurrents.tol.cdf8b74881f87e3b01d56b43791626d2. 10.1371/currents.tol.cdf8b74881f87e3b01d56b43791626d2. 24987576 PMC4073882

[ele70089-bib-0011] Burridge, A. K. , E. Goetze , N. Raes , J. Huisman , and K. T. C. A. Peijnenburg . 2015. “Global Biogeography and Evolution of Cuvierina Pteropods.” BMC Evolutionary Biology 15, no. 1: 39. 10.1186/s12862-015-0310-8. 25880735 PMC4443520

[ele70089-bib-0012] Burridge, A. K. , R. Van Der Hulst , E. Goetze , and K. T. C. A. Peijnenburg . 2019. “Assessing Species Boundaries in the Open Sea: An Integrative Taxonomic Approach to the Pteropod Genus Diacavolinia.” Zoological Journal of the Linnean Society 187, no. 4: 1016–1040. 10.1093/zoolinnean/zlz049.

[ele70089-bib-0013] Chivers, W. J. , A. W. Walne , and G. C. Hays . 2017. “Mismatch Between Marine Plankton Range Movements and the Velocity of Climate Change.” Nature Communications 8, no. 1: 14434. 10.1038/ncomms14434. PMC5309926 28186097

[ele70089-bib-0014] Choo, L. Q. , G. Spagliardi , M. Malinsky , et al. 2023. “Genome‐Wide Phylogeography Reveals Cryptic Speciation in the Circumglobal Planktonic Calcifier *Limacina bulimoides* .” Molecular Ecology 32, no. 12: 3200–3219. 10.1111/mec.16931. 36943181

[ele70089-bib-0015] Choo, L. Q. , T. M. P. Bal , E. Goetze , and K. T. C. A. Peijnenburg . 2021. “Oceanic Dispersal Barriers in a Holoplanktonic Gastropod.” Journal of Evolutionary Biology 34, no. 1: 224–240. 10.1111/jeb.13735. 33150701 PMC7894488

[ele70089-bib-0016] Colwell, R. K. , and T. F. Rangel . 2009. “Hutchinson's Duality: The Once and Future Niche.” Proceedings of the National Academy of Sciences 106, no. supplement_2: 19651–19658. 10.1073/pnas.0901650106. PMC2780946 19805163

[ele70089-bib-0017] da Silva, D. , A. E. Aires , J. P. Zurano , M. A. Olalla‐Tárraga , and P. A. Martinez . 2020. “Changing Only Slowly: The Role of Phylogenetic Niche Conservatism in Caviidae (Rodentia) Speciation.” Journal of Mammalian Evolution 27, no. 4: 713–721. 10.1007/s10914-020-09501-0.

[ele70089-bib-0018] Dam, H. G. 2013. “Evolutionary Adaptation of Marine Zooplankton to Global Change.” Annual Review of Marine Science 5: 349–370. 10.1146/annurev-marine-121211-172229. 22809192

[ele70089-bib-0019] Dam, H. G. , J. A. deMayo , G. Park , et al. 2021. “Rapid, but Limited, Zooplankton Adaptation to Simultaneous Warming and Acidification.” Nature Climate Change 11, no. 9: 780–786. 10.1038/s41558-021-01131-5.

[ele70089-bib-0020] Dawson, M. N. , and D. K. Jacobs . 2001. “Molecular Evidence for Cryptic Species of *Aurelia aurita* (Cnidaria, Scyphozoa).” Biological Bulletin 200, no. 1: 92–96. 10.2307/1543089. 11249217

[ele70089-bib-0021] Dormann, C. F. , J. Elith , S. Bacher , et al. 2013. “Collinearity: A Review of Methods to Deal With It and a Simulation Study Evaluating Their Performance.” Ecography 36, no. 1: 27–46. 10.1111/j.1600-0587.2012.07348.x.

[ele70089-bib-0022] Goetze, E. 2005. “Global Populatiion Genetic Structure and Biogeography of the Oceanic Copepods *Eucalanus hyalinus* and *e. Spinifer* .” Evolution 59, no. 11: 2378–2398. 10.1111/j.0014-3820.2005.tb00948.x. 16396179

[ele70089-bib-0023] Goetze, E. 2003. “Cryptic Speciation on the High Seas; Global Phylogenetics of the Copepod Family Eucalanidae.” Proceedings of the Royal Society B: Biological Sciences 270, no. 1531: 2321–2331. 10.1098/rspb.2003.2505. PMC1691510 14667347

[ele70089-bib-0024] Goetze, E. 2011. “Population Differentiation in the Open Sea: Insights From the Pelagic Copepod *Pleuromamma xiphias* .” Integrative and Comparative Biology 51, no. 4: 580–597. 10.1093/icb/icr104. 21940778

[ele70089-bib-0025] Goetze, E. , P. T. Hüdepohl , C. Chang , L. Van Woudenberg , M. Iacchei , and K. T. C. A. Peijnenburg . 2017. “Ecological Dispersal Barrier Across the Equatorial Atlantic in a Migratory Planktonic Copepod.” Progress in Oceanography 158: 203–212. 10.1016/j.pocean.2016.07.001.

[ele70089-bib-0026] Goetze, E. , and M. D. Ohman . 2010. “Integrated Molecular and Morphological Biogeography of the Calanoid Copepod Family Eucalanidae.” Deep Sea Research Part II: Topical Studies in Oceanography 57, no. 24–26: 2110–2129. 10.1016/j.dsr2.2010.09.014.

[ele70089-bib-0027] González, C. E. , E. Goetze , R. Escribano , O. Ulloa , and P. Victoriano . 2020. “Genetic Diversity and Novel Lineages in the Cosmopolitan Copepod *Pleuromamma abdominalis* in the Southeast Pacific.” Scientific Reports 10, no. 1: 1115. 10.1038/s41598-019-56935-5. 31980660 PMC6981114

[ele70089-bib-0028] Guisan, A. , W. Thuiller , and N. E. Zimmermann . 2017. Habitat Suitability and Distribution Models: With Applications in R. Cambridge University Press.

[ele70089-bib-0029] Hays, G. C. , A. J. Richardson , and C. Robinson . 2005. “Climate Change and Marine Plankton.” Trends in Ecology & Evolution 20, no. 6: 337–344. 10.1016/j.tree.2005.03.004. 16701390

[ele70089-bib-0030] Hinder, S. L. , M. B. Gravenor , M. Edwards , et al. 2014. “Multi‐Decadal Range Changes vs. Thermal Adaptation for North East Atlantic Oceanic Copepods in the Face of Climate Change.” Global Change Biology 20, no. 1: 140–146. 10.1111/gcb.12387. 24323534

[ele70089-bib-0031] Hirai, J. , A. Tsuda , and E. Goetze . 2015. “Extensive Genetic Diversity and Endemism Across the Global Range of the Oceanic Copepod *Pleuromamma abdominalis* .” Progress in Oceanography 138: 77–90. 10.1016/j.pocean.2015.09.002.

[ele70089-bib-0032] Hirzel, A. H. , G. Le Lay , V. Helfer , C. Randin , and A. Guisan . 2006. “Evaluating the Ability of Habitat Suitability Models to Predict Species Presences.” Ecological Modelling 199, no. 2: 142–152. 10.1016/j.ecolmodel.2006.05.017.

[ele70089-bib-0033] Hu, J. , Z. Jiang , J. Chen , and H. Qiao . 2015. “Niche Divergence Accelerates Evolution in Asian Endemic Procapra Gazelles.” Scientific Reports 5, no. 1: 10069. 10.1038/srep10069. 25951051 PMC4423425

[ele70089-bib-0034] Hua, X. , and J. J. Wiens . 2010. “Latitudinal Variation in Speciation Mechanisms in Frogs.” Evolution 64, no. 2: 429–443. 10.1111/j.1558-5646.2009.00836.x. 19744118

[ele70089-bib-0035] Hutchinson, G. E. 1957. “The Multivariate Niche.” In Cold Spring Harbor Symposia on Quantitative Biology, vol. 22, 415–421. Paper Presented at the Annual Conference, 1933–2003.

[ele70089-bib-0036] Jarman, S. N. , N. G. Elliott , S. Nicol , and A. McMinn . 2002. “Genetic Differentiation in the Antarctic Coastal Krill *Euphausia crystallorophias* .” Heredity 88, no. 4: 280–287. 10.1038/sj.hdy.6800041. 11920136

[ele70089-bib-0037] Johnston, N. M. , E. J. Murphy , A. Atkinson , et al. 2022. “Status, Change, and Futures of Zooplankton in the Southern Ocean.” Frontiers in Ecology and Evolution 9: 624692. 10.3389/fevo.2021.624692.

[ele70089-bib-0038] Jönsson, B. F. , and J. R. Watson . 2016. “The Timescales of Global Surface‐Ocean Connectivity.” Nature Communications 7, no. 1: 11239. 10.1038/ncomms11239. PMC4838858 27093522

[ele70089-bib-0039] Laso‐Jadart, R. , M. O'Malley , A. M. Sykulski , C. Ambroise , and M. A. Madoui . 2023. “Holistic View of the Seascape Dynamics and Environment Impact on Macro‐Scale Genetic Connectivity of Marine Plankton Populations.” BMC Ecology and Evolution 23, no. 1: 46. 10.1186/s12862-023-02160-8. 37658324 PMC10472650

[ele70089-bib-0040] Lobo, J. M. , A. Jiménez‐Valverde , and J. Hortal . 2010. “The Uncertain Nature of Absences and Their Importance in Species Distribution Modelling.” Ecography 33, no. 1: 103–114. 10.1111/j.1600-0587.2009.06039.x.

[ele70089-bib-0041] McCormack, J. E. , A. J. Zellmer , and L. L. Knowles . 2010. “Does Niche Divergence Accompany Allopatric Divergence in Aphelocoma Jays as Predicted Under Ecological Speciation?: Insights From Tests With Niche Models.” Evolution 64, no. 5: 1231–1244. 10.1111/j.1558-5646.2009.00900.x. 19922442

[ele70089-bib-0042] McGinty, N. , A. D. Barton , Z. V. Finkel , D. G. Johns , and A. J. Irwin . 2021. “Niche Conservation in Copepods Between Ocean Basins.” Ecography 44, no. 11: 1653–1664. 10.1111/ecog.05690.

[ele70089-bib-0043] Murphy, E. J. , N. M. Johnston , E. E. Hofmann , et al. 2021. “Global Connectivity of Southern Ocean Ecosystems.” Frontiers in Ecology and Evolution 9: 624451. 10.3389/fevo.2021.624451.

[ele70089-bib-0044] Nelson, R. J. , E. C. Carmack , F. McLaughlin , and G. Cooper . 2009. “Penetration of Pacific Zooplankton Into the Western Arctic Ocean Tracked With Molecular Population Genetics.” Marine Ecology Progress Series 381: 129–138. 10.3354/meps07940.

[ele70089-bib-0045] Norris, R. D. 2000. “Pelagic Species Diversity, Biogeography, and Evolution.” Paleobiology 26, no. S4: 236–258. 10.1017/S0094837300026956.

[ele70089-bib-0046] Norton, E. L. , and E. Goetze . 2013. “Equatorial Dispersal Barriers and Limited Population Connectivity Among Oceans in a Planktonic Copepod.” Limnology and Oceanography 58, no. 5: 1581–1596. 10.4319/lo.2013.58.5.1581.

[ele70089-bib-0047] Pearman, P. B. , A. Guisan , O. Broennimann , and C. F. Randin . 2008. “Niche Dynamics in Space and Time.” Trends in Ecology & Evolution 23, no. 3: 149–158. 10.1016/j.tree.2007.11.005. 18289716

[ele70089-bib-0048] Pedersen, E. J. , D. L. Miller , G. L. Simpson , and N. Ross . 2019. “Hierarchical Generalized Additive Models in Ecology: An Introduction With mgcv.” PeerJ 7: e6876. 10.7717/peerj.6876. 31179172 PMC6542350

[ele70089-bib-0049] Peijnenburg, K. T. C. A. , C. Fauvelot , J. A. J. Breeuwer , and S. B. J. Menkine . 2006. “Spatial and Temporal Genetic Structure of the Planktoni Csagitta Setosa (Chaetognatha) in European Seas as Revealed by Mitochondrial and Nuclear DNA Markers.” Molecular Ecology 15, no. 11: 3319–3338. 10.1111/j.1365-294X.2006.03002.x. 16968273

[ele70089-bib-0050] Peijnenburg, K. T. C. A. , and E. Goetze . 2013. “High Evolutionary Potential of Marine Zooplankton.” Ecology and Evolution 3, no. 8: 2765–2781. 10.1002/ece3.644. 24567838 PMC3930040

[ele70089-bib-0051] Peterson, A. T. 2011. “Ecological Niche Conservatism: A Time‐Structured Review of Evidence: Ecological Niche Conservatism.” Journal of Biogeography 38, no. 5: 817–827. 10.1111/j.1365-2699.2010.02456.x.

[ele70089-bib-0052] Petitpierre, B. , C. Kueffer , O. Broennimann , C. Randin , C. Daehler , and A. Guisan . 2012. “Climatic Niche Shifts Are Rare Among Terrestrial Plant Invaders.” Science 335, no. 6074: 1344–1348. 10.1126/science.1215933. 22422981

[ele70089-bib-0053] Phillips, S. J. , R. P. Anderson , and R. E. Schapire . 2006. “Maximum Entropy Modeling of Species Geographic Distributions.” Ecological Modelling 190, no. 3–4: 231–259. 10.1016/j.ecolmodel.2005.03.026.

[ele70089-bib-0054] Pinsky, M. L. , A. M. Eikeset , D. J. McCauley , J. L. Payne , and J. M. Sunday . 2019. “Greater Vulnerability to Warming of Marine Versus Terrestrial Ectotherms.” Nature 569, no. 7754: 108–111. 10.1038/s41586-019-1132-4. 31019302

[ele70089-bib-0055] Provan, J. , G. E. Beatty , S. L. Keating , C. A. Maggs , and G. Savidge . 2009. “High Dispersal Potential Has Maintained Long‐Term Population Stability in the North Atlantic Copepod *Calanus finmarchicus* .” Proceedings of the Royal Society B: Biological Sciences 276, no. 1655: 301–307. 10.1098/rspb.2008.1062. PMC2674349 18812293

[ele70089-bib-0056] Richter, D. J. , R. Watteaux , T. Vannier , et al. 2022. “Genomic Evidence for Global Ocean Plankton Biogeography Shaped by Large‐Scale Current Systems.” eLife 11: e78129. 10.7554/eLife.78129. 35920817 PMC9348854

[ele70089-bib-0057] Romdal, T. S. , M. B. Araújo , and C. Rahbek . 2013. “Life on a Tropical Planet: Niche Conservatism and the Global Diversity Gradient.” Global Ecology and Biogeography 22, no. 3: 344–350. 10.1111/j.1466-8238.2012.00786.x.

[ele70089-bib-0058] Sasaki, M. , J. M. Barley , S. Gignoux‐Wolfsohn , et al. 2022. “Greater Evolutionary Divergence of Thermal Limits Within Marine Than Terrestrial Species.” Nature Climate Change 12, no. 12: 1175–1180. 10.1038/s41558-022-01534-y.

[ele70089-bib-0059] Sasaki, M. C. , and H. G. Dam . 2019. “Integrating Patterns of Thermal Tolerance and Phenotypic Plasticity With Population Genetics to Improve Understanding of Vulnerability to Warming in a Widespread Copepod.” Global Change Biology 25, no. 12: 4147–4164. 10.1111/gcb.14811. 31449341

[ele70089-bib-0060] Sasaki, M. , and H. G. Dam . 2021. “Global Patterns in Copepod Thermal Tolerance.” Journal of Plankton Research 43, no. 4: 598–609. 10.1093/plankt/fbab044.

[ele70089-bib-0061] Schlueter, M. H. , A. Merico , M. Reginatto , M. Boersma , K. H. Wiltshire , and W. Greve . 2010. “Phenological Shifts of Three Interacting Zooplankton Groups in Relation to Climate Change.” Global Change Biology 16, no. 11: 3144–3153.

[ele70089-bib-0062] Smith, A. B. , W. Godsoe , F. Rodríguez‐Sánchez , H.‐H. Wang , and D. Warren . 2019. “Niche Estimation Above and Below the Species Level.” Trends in Ecology & Evolution 34, no. 3: 260–273. 10.1016/j.tree.2018.10.012. 30497791

[ele70089-bib-0063] Soberón, J. 2007. “Grinnellian and Eltonian Niches and Geographic Distributions of Species.” Ecology Letters 10, no. 12: 1115–1123. 10.1111/j.1461-0248.2007.01107.x. 17850335

[ele70089-bib-0064] Soberón, J. , and A. T. Peterson . 2020. “What Is the Shape of the Fundamental Grinnellian Niche?” Theoretical Ecology 13, no. 1: 105–115. 10.1007/s12080-019-0432-5.

[ele70089-bib-0065] Sommeria‐Klein, G. , R. Watteaux , F. M. Ibarbalz , et al. 2021. “Global Drivers of Eukaryotic Plankton Biogeography in the Sunlit Ocean.” Science 374, no. 6567: 594–599. 10.1126/science.abb3717. 34709919

[ele70089-bib-0066] Stupnikova, A. N. , T. N. Molodtsova , N. S. Mugue , and T. V. Neretina . 2013. “Genetic Variability of the *Metridia lucens* Complex (Copepoda) in the Southern Ocean.” Journal of Marine Systems 128: 175–184. 10.1016/j.jmarsys.2013.04.016.

[ele70089-bib-0067] Valavi, R. , J. Elith , J. J. Lahoz‐Monfort , and G. Guillera‐Arroita . 2023. “Flexible Species Distribution Modelling Methods Perform Well on Spatially Separated Testing Data.” Global Ecology and Biogeography 32, no. 3: 369–383. 10.1111/geb.13639.

[ele70089-bib-0068] Villarino, E. , G. Chust , P. Licandro , et al. 2015. “Modelling the Future Biogeography of North Atlantic Zooplankton Communities in Response to Climate Change.” Marine Ecology Progress Series 531: 121–142. 10.3354/meps11299.

[ele70089-bib-0069] Villarino, E. , X. Irigoien , F. Villate , et al. 2020. “Response of Copepod Communities to Ocean Warming in Three Time‐Series Across the North Atlantic and Mediterranean Sea.” Marine Ecology Progress Series 636: 47–61. 10.3354/meps13209.

[ele70089-bib-0070] Villarino, E. , J. R. Watson , B. Jönsson , et al. 2018. “Large‐Scale Ocean Connectivity and Planktonic Body Size.” Nature Communications 9, no. 1: 142. 10.1038/s41467-017-02535-8. PMC5762663 29321528

[ele70089-bib-0071] Warren, D. L. , R. E. Glor , and M. Turelli . 2008. “Environmental Niche Equivalency Versus Conservatism: Quantitative Approaches to Niche Evolution.” Evolution 62, no. 11: 2868–2883. 10.1111/j.1558-5646.2008.00482.x. 18752605

[ele70089-bib-0072] Weydmann, A. , N. C. Coelho , E. A. Serrao , A. Burzyński , and G. A. Pearson . 2016. “Pan‐Arctic Population of the Keystone Copepod *Calanus glacialis* .” Polar Biology 39: 2311–2318. 10.1007/s00300-016-1898-x.

[ele70089-bib-0073] Wiens, J. J. , D. D. Ackerly , A. P. Allen , et al. 2010. “Niche Conservatism as an Emerging Principle in Ecology and Conservation Biology.” Ecology Letters 13, no. 10: 1310–1324. 10.1111/j.1461-0248.2010.01515.x. 20649638

[ele70089-bib-0074] Wiens, J. J. , and C. H. Graham . 2005. “Niche Conservatism: Integrating Evolution, Ecology, and Conservation Biology.” Annual Review of Ecology, Evolution, and Systematics 36, no. 1: 519–539. 10.1146/annurev.ecolsys.36.102803.095431.

[ele70089-bib-0075] Zurell, D. , J. Franklin , C. König , et al. 2020. “A Standard Protocol for Reporting Species Distribution Models.” Ecography 43, no. 9: 1261–1277. 10.1111/ecog.04960.

