# Supplementary material for: Global Variation in Zooplankton Niche Divergence Across Ocean Basins
Source: Ecol Lett. 2025 Feb 20;28(2):e70089. doi: 10.1111/ele.70089 (PMC11841027; doi:10.1111/ele.70089)
Supplement: Supplementary file 1 — Appendix S1. [file ELE-28-0-s001.docx]

**Supplementary figures and tables for: Global variation in zooplankton niche divergence across ocean basins**

**Authors:** Niall McGinty^1^, Andrew Irwin^2^

1. Department of Oceanography, Dalhousie University, Halifax, Canada
2. Department of Mathematics & Statistics, Dalhousie University, Halifax, Canada

**Corresponding author:** nmcginty@dal.ca

**Supplementary Figure 1. A) Distribution of sampling effort globally aggregated into 1x1 degree cells. Warmer colours indicate areas with a greater number of observations. B) A table showing the number of observations for the main taxonomic groups within each of the seven ocean basins.**

**
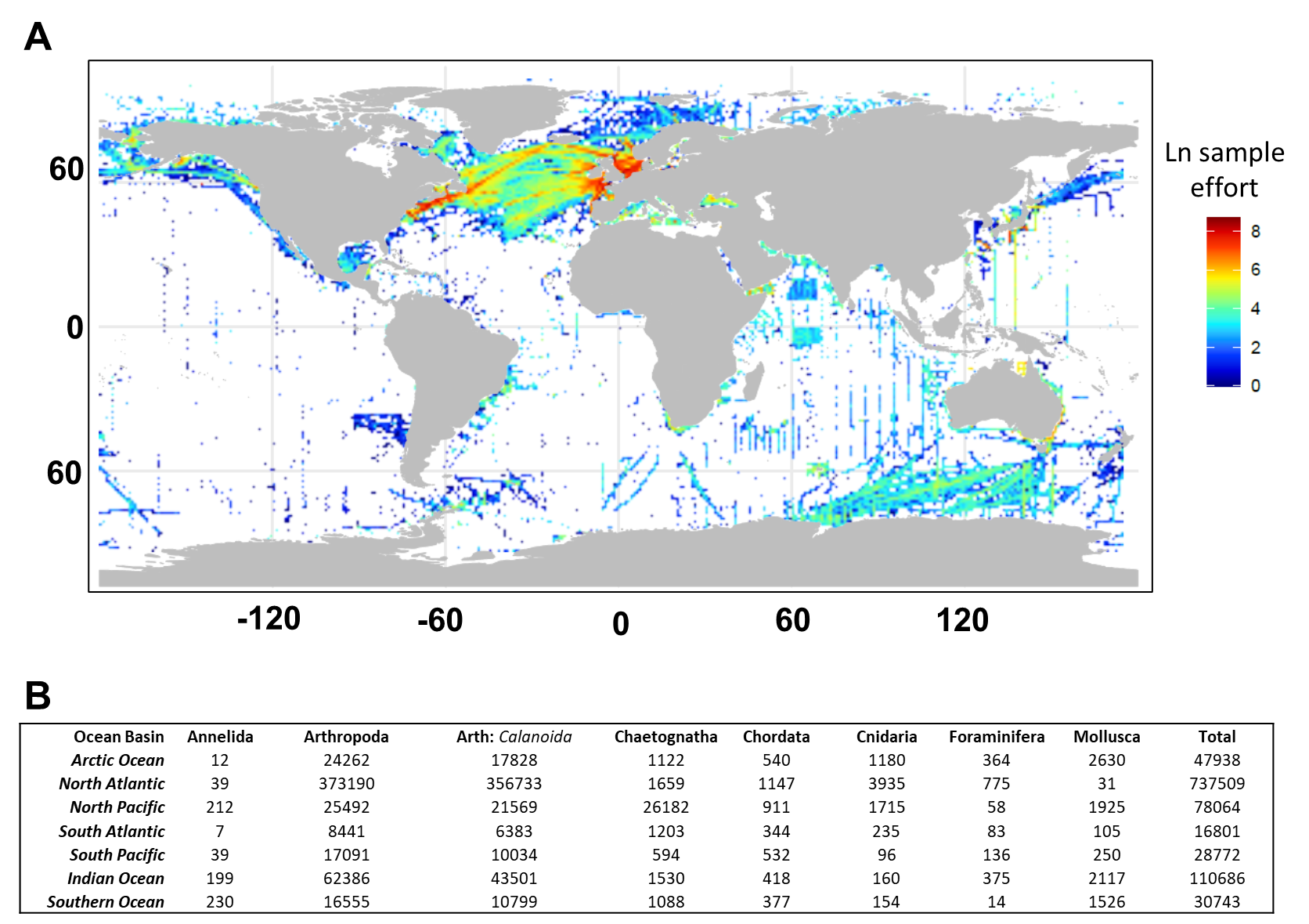
**

**Supplementary Figure 2: The spatial clustering of the zooplankton presences using the Jaccard similarity index and hierarchical agglomerative clustering using A) Data aggregated into 1x1 degree grid and B) Averaged within each of the Longhurst biogeochemical provinces. C) The Haversine distance (Interquartile range) in km between the species observations within each of the seven clusters defined by the Longhurst provinces along with the global distances across all clusters. D) The Haversine distance (Interquartile range) in km between the species observations within each of the seven ocean basins along with the global distances across all ocean basins.**

**
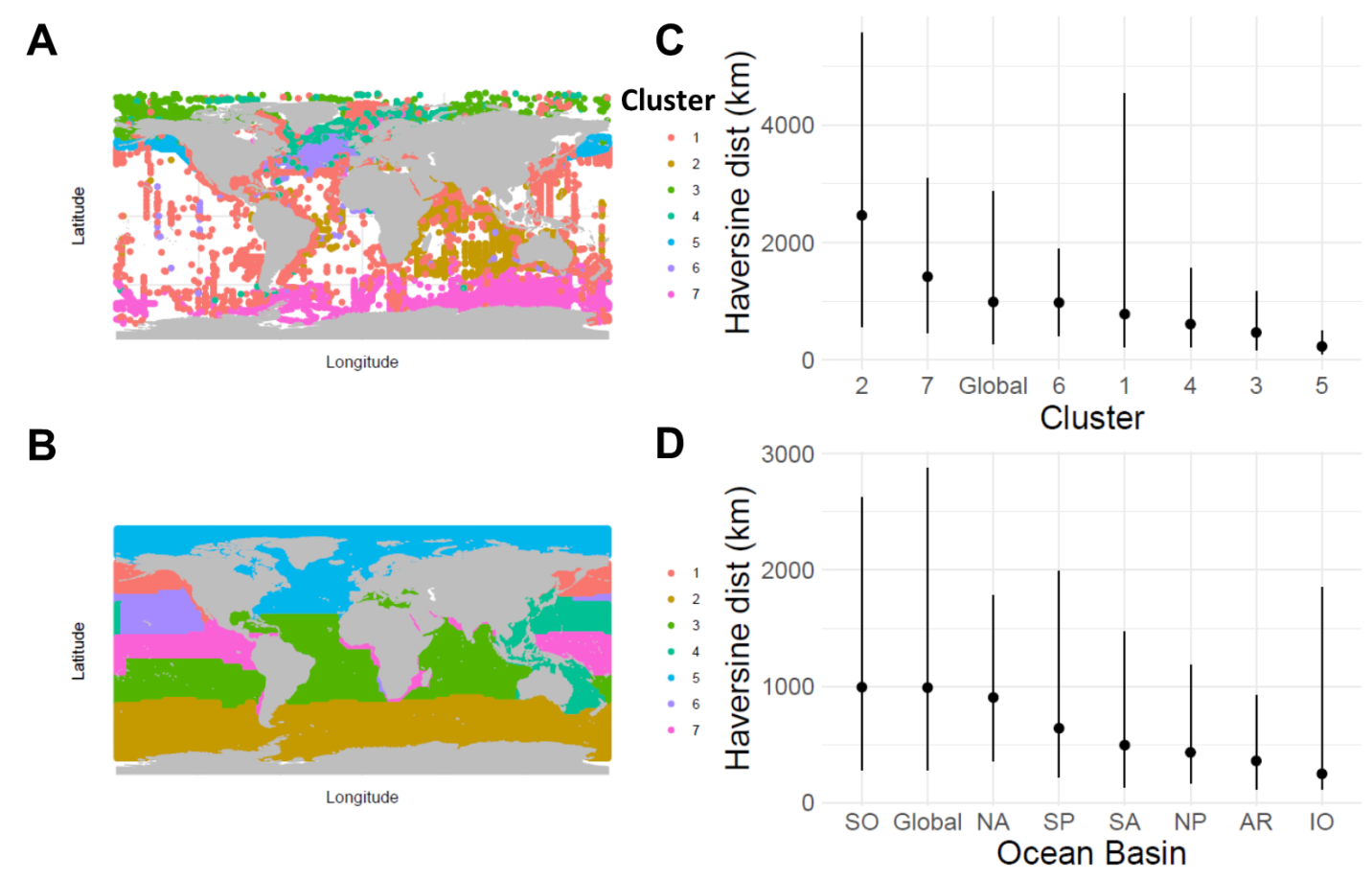
**

**Supplementary Figure 3. The and Continuous Boyce Index (CBI; upper panels) and true skill statistic (TSS; lower panels) used for evaluating each of the three environmental niche model (ENM) algorithms in the ensemble (GLM – Generalised additive model; GAM – Generalised additive model; MAXENT – Maximum entropy). The mean and confidence interval for the main taxonomic groups are shown.**

**
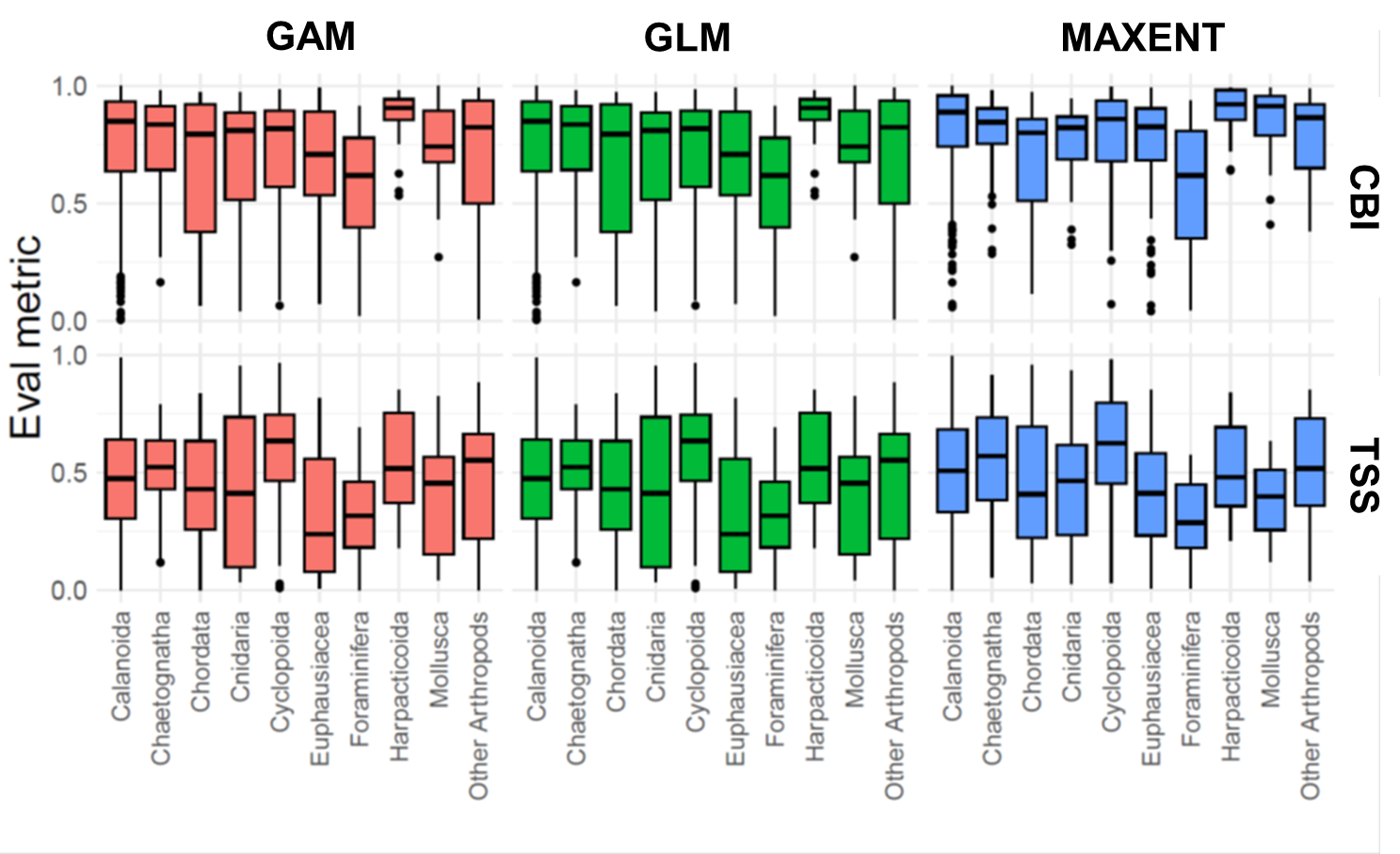
**

**Supplementary Figure 4. Variable importance per Taxonomic group (ensemble). A) Calanoida; B) Chaetognatha; C) Chordata; D) Cnidaria; E) Cyclopoida; G) Euphausiacea; H) Foraminifera; I) Mollusca; J) Other Arthropods**

**
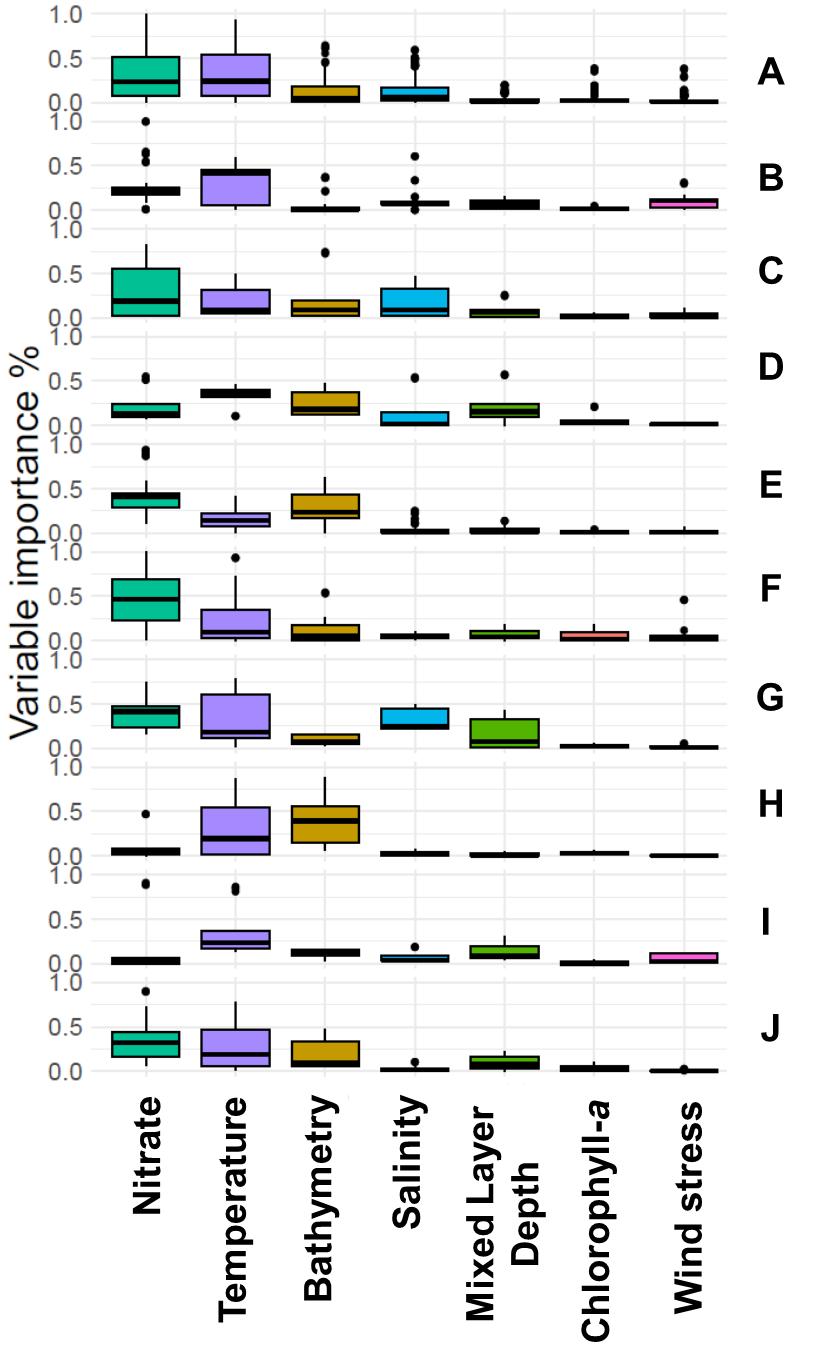
**

**Supplementary Figure 5. A density plot of the environmental conditions for each of the seven ocean basins labelled using their 2 charachter code and coloured according (see Figure 1). The density plots for A) temperature, B) log chlorophyll-a, C) Salinity, D) mixed layer depth, E) wind stress, F) nitrate and G) bathymetry**

**
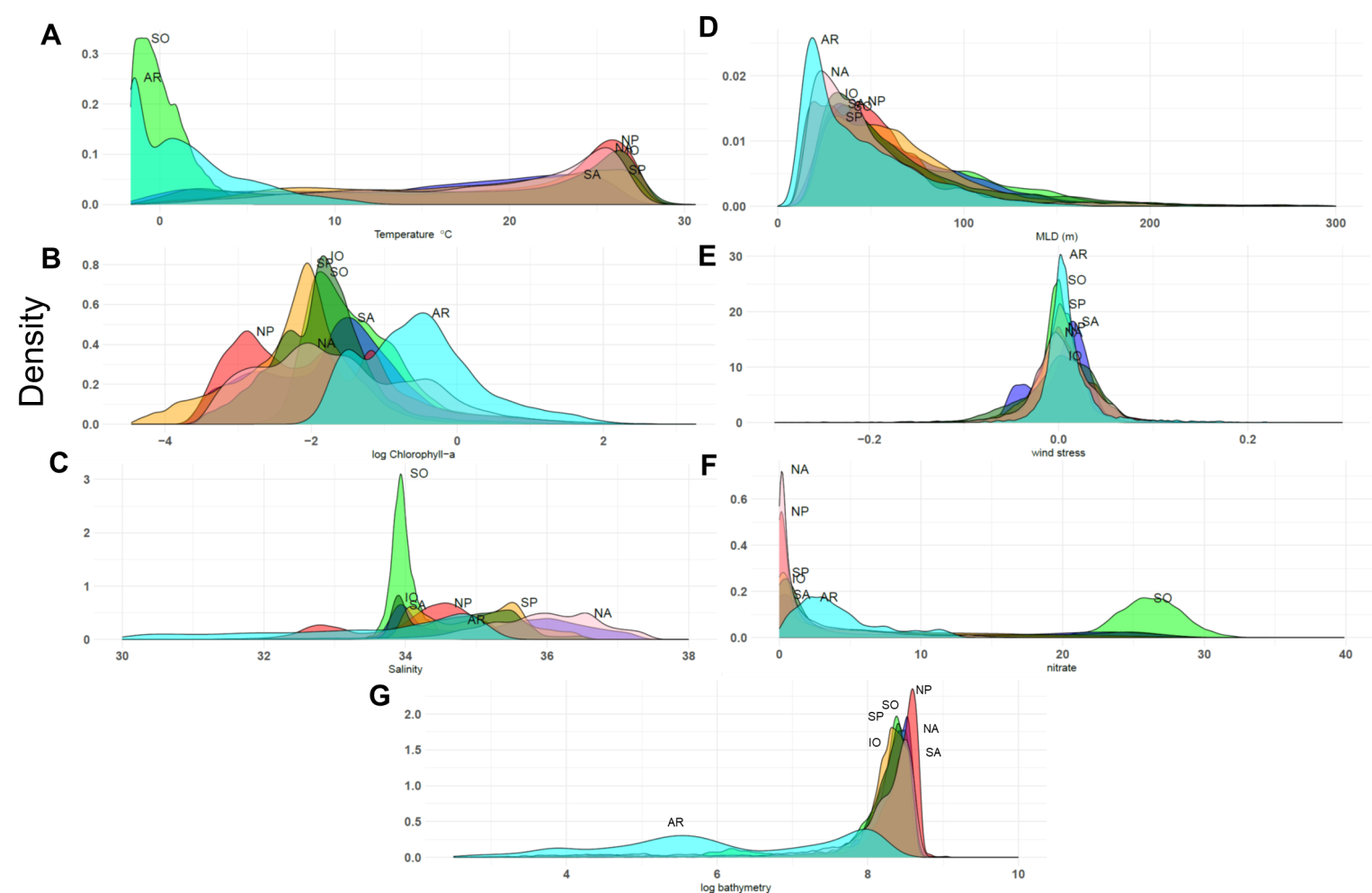
**

**Supplementary Figure 6. The difference in Schoener’s D (Delta D) niche overlap for using the original observation number for two paired-area observations and the resampled number ranging from 25 – 1000 for seven increasing steps in number. The ribbon plot shows the spread in the Delta D for each resampling number while the black point shows the median delta D for that sampling number. Also shown are the changes between each resampling number level for the 829 paired-area comparisons.**

**Supplementary Table 1:** The list of the 8 covariates used constructing the environmental niche ensemble models. Shown are the variables with units in parenthesis, the source and location of each variable and the spatial and temporal input resolution of the original data.

| **Variable (units)** | **Source** | **Location** | **Input resolution** |
| --- | --- | --- | --- |
| Sea temperature (°C) | World Ocean Atlas 2023 | https://www.ncei.noaa.gov/products/world-ocean-atlas | Monthly 1° |
| Salinity (unitless) | World Ocean Atlas 2023 | https://www.ncei.noaa.gov/products/world-ocean-atlas | Monthly 1° |
| Nitrate (µmol kg^-1^) | World Ocean Atlas 2023 | https://www.ncei.noaa.gov/products/world-ocean-atlas | Monthly 1° |
| Mixed Layer Depth (m) | World Ocean Atlas 2023 | https://www.ncei.noaa.gov/products/world-ocean-atlas | Monthly 1° |
| Dissolved Oxygen ml l^-1^ | World Ocean Atlas 2023 | https://www.ncei.noaa.gov/products/world-ocean-atlas | Monthly 1° |
| Bathymetry (m) | GEBCO 2023 | https://www.gebco.net/ | NA 0.16° |
| Chlorophyll a (mg m ^−3^) | GlobColour | https://www.globcolour.info/ | Monthly 9km^2^ |
| Wind stress (N m^-2^) | AVISO | https://www.aviso.altimetry.fr/en/home.html | Weekly 0.25° |

**Supplementary Table 2: See excel file – An overview of the 829 paired-area comparisons. Descriptions of each column are included with the document.**

**Supplementary Table 3: See excel file – The document that outlines our ODMAP (Overview, Data, Model, Assessment and Prediction) protocol for our environmental niche model ensembles.**
