# Supplementary material for: Global Variation in Zooplankton Niche Divergence Across Ocean Basins
Source: Ecol Lett. 2025 Feb 20;28(2):e70089. doi: 10.1111/ele.70089 (PMC11841027; doi:10.1111/ele.70089)
Supplement: Supplementary file 4 — Note S1. [file ELE-28-0-s004.docx]

**Supplementary Note 1: Global variation in zooplankton niche divergence across ocean basins**

**Authors:** Niall McGinty^1^, Andrew Irwin^2^

1. Department of Oceanography, Dalhousie University, Halifax, Canada
2. Department of Mathematics & Statistics, Dalhousie University, Halifax, Canada

**Corresponding author:** nmcginty@dal.ca

**Material and methods**

Our study aims to investigate the potential for niche divergence of globally distributed marine meso-zooplankton between the seven main ocean basins. For each species we develop a global environmental niche model (ENM) using an ensemble of three different algorithms which cover machine learning and linear/non-linear methods. The global ensemble ENM is used in two ways. Firstly, we use the ENM to understand the mean niche of each species in order to define where each species fits along each environmental gradient used in the ENM. For example, high temperature niche suggests a tropical affinity species and *vice versa*. Secondly, the model weights of the three algorithms used in the ensemble are used for the niche divergence tests where paired groups of species observations within different ocean basins are compared. Finally, we look for potential environmental signals in the level of niche divergence for species that belong to the Class Copepoda. We focus on this group due to their ubiquity within the dataset (~70%) and availability of observations within the paired ocean basins. All data processing and analyses were performed using the R programming environment (ver. 4.3.3 ). The packages used for each stage are listed in the appropriate sections below. A schematic representation of the steps used in the analysis is also shown here and in Figure 1 of the main manuscript.

**Zooplankton data**

Global zooplankton observations were extracted from the “Zoobase” database (Benedetti et al., 2021). The database consists of observations collated from the OBIS and GBIF repositories for the main taxonomic groups that comprise the bulk of global mesozooplankton biomass. A spatial filtering was applied following Benedetti et al. (2023) to reduce bias caused by sampling effort. Data were first limited to occurrences collected in the upper 200m and were subsequently thinned such that for each month there is a single observation for each 1° grid. Species with at least 50 observations in at least two of the seven ocean basins were retained for the niche divergence analysis (see section on ***robustness tests*** and ***study area*** below). In total we found 267 species that were present in sufficient numbers from at least two ocean basins resulting in 789,231 unique observations.

**Biotic variable selection**

We selected a set of biotic and abiotic variables that have been demonstrated to influence the global distribution of zooplankton either directly though controlling metabolic processes (temperature) or indirectly by serving as a proxy for food availability (chlorophyll-*a*) (Supp. Table 1). The R packages *raster* and *ncdf4* were primarily used to extract the data from these variables. Data from the list of candidate variables were extracted into monthly climatologies with a 1° resolution collected between 1955-2022 from the World Ocean Atlas (WOA, 2023). Average values for the upper 200m were used for sea temperature (°C), salinity, nitrates; (NO3-), and dissolved oxygen concentration (ml l^-1^). Also included was the mixed layer depth (MLD; m). Monthly values of wind stress (m^2^ s^−2^) with 0.25^○^ resolution was obtained from the Aviso dataset (see the Aviso website; [www.aviso.altimetry.fr](http://www.aviso.altimetry.fr) which serves as a proxy for sub-mesoscale dynamics and eddy formation. Monthly values of chlorophyll-*a* (chl-*a* mg m^−3^) which serve as a proxy for primary productivity biomass were derived from merged monthly satellite products from the GlobColour database between 1998-2022 with a grid size of 9 km^2^ (globcolour.info). Bathymetric depth (m) served as a proxy for onshore/offshore affinity and was extracted from the GEBCO topographical dataset with (1’) spatial resolution (GEBCO, 2023). Bathymetry, wind stress and Chl-*a* data were aggregated and averaged to match the 1^○^ resolution and monthly climatologies of the WOA (Supplementray Table 1).

The choice of environmental predictors from the list of candidate variables was examined for an imbalance in sampling effort globally and potential multicollinearity or autocorrelated variables. For variable imbalance we examine the the imabalance of sampling effort of sampling space for all species with greater than 100 observations. We find that salinity has the greatest observational imbalance, particularly due to the lower salinity values found in the Baltic/Black seas that are not found elsewhere. Instead of removing the salinity variables, we choose to remove the marginal seas from the analysis and focus on the larger open ocean basins (See study area below). multicollinearity was assessed for the list of candidate abiotic and biotic variables using a Spearman correlation and the variance inflation factor (VIF) using a cut-off of r < 0.75 and VIF < 5 for variables to be included (Dormann et al., 2013). Only temperature and dissolved oxygen concentration were highly correlated (r = 0.91, VIF = 17.5) based on the selection criteria and as a result dissolved oxygen concentration was removed from the list of candidate variables. The remaining candidate variables of temperature, salinity, nitrate, MLD, chl-*a*, wind stress and bathymetry were retained for modeling. The zooplankton data were linked to the environmental data by matching observations co-located within the same month and 1° grid of the environmental data.

**Study area**

Ocean basins are both a natural construct and, an artificial one, depending on how we define them. The world’s oceans are interconnected, so the concept of distinct "ocean basins" often arises from human categorization for convenience in understanding and studying marine geography. However, there are also **natural barriers** and features that differentiate these basins, influencing water movement and interconnectivity of marine species. We first attempted to look at the differences in community composition for the data to determine potential ecoregions in order to classify different regions of the ocean. We used our database of 224 species to create a presence/absence dataset at a 1° resolution globally. We performed a PERMANOVA using the Jaccard distance for species occurrence with 999 permutations within the package *vegan*. Jaccard distance measures the dissimilarity between grid cells by dividing the number of common species by the total unique number of species in both cells. It is useful for differentiating the gradients in species turnover and is robust index for minimising sampling error (Schroeder & Jenkins, 2018). The resulting clusters were separated into 7 clusters based on the silhouette method and examined spatially. We found that the clusters were not spatially coherent and some had distributions that spanned across multiple ocean basins (See Supplementary Figure 2). As we are interested in spatial distances between observations or groups of observations we want to minimise the within-group distance between observations compared to between-groups of observations. The presence of the same cluster separated by continental landmasses in contrast with others significantly impacts the distances between observations. A comparison of the geographical distance between species presences within ocean basins were consistently less than those across ocean basins while the geographical distances within clusters varied As a result we restricted our observations and comparisons to groups within the seven major ocean basins (North Atlantic, South Atlantic, North Pacific, South Pacific, Indian, Arctic and Southern Oceans). We restrict our observations to these main ocean basins and excluding many of the marginal seas which include the Mediterranean, Black and Baltic seas due to the imbalances of salinity values in these areas compared to others. Our final dataset consists of 224 unique zooplankton species from 642,364 observations.

**Environmental niche model ensembles (ENM)**

The first stage involves constructing a global ENM ensemble, collating all records of each species globally using the seven abiotic and biotic variables retained after testing for multicollinearity. Each of the 224 species contained at least 100 observations (i.e - 50 observations in two or more ocean basins) which maintained a relatively high presence to predictor ratio (100 observations and 7 predictors) which is recommended to be at least 10 (Guisan et al., 2017).

We use the *biomod2* package in R to create an ensemble model for ENM integrating predictions across three multiple modeling algorithms, Maximum entropy (MaxEnt), Generalized Linear Models (GLM), and Generalized Additive Models (GAM). Ensemble models, which combine outputs from different algorithms, can improve prediction accuracy and reduce uncertainty by taking advantage of each algorithm’s strengths. While other algorithms are available we focus on these as they are primarily used when testing for niche overlap and represent a range of machine learning, linear and non-linear methods for estimating a species distribution.

We restricted each MaxEnt model to ‘default’ settings, limiting the regularisation multiplier to 1 and transformations of input predictors beyond linear or quadratic (Valavi et al., 2023) and use the same settings for all models to avoid biases that could be introduced through model interactions in an ensemble or different parameterisation of each MaxEnt model (Barber et al., 2022). For the GLM, we use a logistic link function (presence/absence) and restrict the formula to not include any interactions between variables to prevent over fitting. The GAM which is an extension of GLM, allows for nonlinear relationships by using smoothing functions on predictors. In GAMs, smoothness is controlled by setting a parameter which defines the maximum degrees of freedom or "knots" for the smooth term. We restrict this number to 4 to prevent over fitting to ensure a meaningful and interpretable model.

The simple randomised selection of background points in generating ENM often leads to biases in the model estimation particularly if the species modelled are restricted in space or the area contains extreme values (Lobo et al., 2010). To overcome this bias, a targeted group approach was used to select background locations (Phillips et al., 2009). Background locations were selected proportional to the density of the spatially filtered zooplankton occurrence data. To maintain the 10:1 background data to presence ratio, species with many presences were subsampled to a limit of 1000 points (Barbet-Massin et al., 2012).

A critical aspect of generating reliable ensemble models is to use robust resampling techniques for model validation. In spatial ecology**, block resampling** is particularly valuable, as it accounts for spatial autocorrelation and ensures that model evaluation metrics reflect the model's true predictive performance across space, rather than just statistical accuracy alone.

Models were evaluated using spatially structured block resampling of the training and testing data where both the presences and background samples are partitioned into different spatial blocks. The block size was determined using the *blockCV* package in R (Valavi et al., 2018) by partitioning using cross-validation of *k* folds (*k* = 5) to allocate a similar number of presences and background points into each fold. The evaluation metrics were computed on the spatially structured training and evaluation datasets using two different metric the True Skill Statistic (TSS) and the continuous Boyce index (CBI) which are better equipped to evaluate presence only data compared with other traditional methods of model evaluation (Hirzel et al., 2006). Both the TSS and CBI are scaled between -1 and 1. The CBI is the Spearman rank correlation coefficient between the proportion of sites in each prediction class and the expected proportion of predictions in each prediction class based on the proportion of the landscape that is in that class. The index ranges from -1 to 1. Values >0 indicate the model's output is positively correlated with the true probability of presence. Values <0 indicate it is negatively correlated with the true probability of presence (Boyce et al., 2002). TSS is calculated by adding sensitivity and specificity together and subtracting 1. Sensitivity is the proportion of presence accurately predicted, and specificity is the proportion of absences accurately predicted. Values < 0 indicate that the model performs no better than random (Allouche et al., 2006). The resulting ENM ensembles for each species were used to extract the mean niche of each environmental gradient. We calculate the weighted mean of each environmental gradient, using the logistic probability of a species presence as the weighting factor. These mean niche values are combined with the niche divergence assessments for further modelling (see below).

**Niche divergence assessed by ENM**

A two-part analysis was used to test for niche conservatism using methods first developed by McCormack et al. (2010) and used to test for niche differences for copepod species that occupy multiple ocean basins (McGinty et al., 2021). The analysis involves using an environmental niche model (ENM) and principal component analysis (PCA) to determine niche conservatism or divergence between ocean basins. Niche divergence is accepted when it is detected from both ENM and PCA methods.

Niche divergence is first assessed by comparing paired area ENM ensembles using the same selection procedures of presence/background data as the global ENM ensembles. We extract presences from two ocean areas and calculate the niche overlap using for each of the three ENM algorithms (MaxEnt, GLM and GAM) using the Schoener’s (D) metric where 0 = no overlap and 1 = perfect overlap. The overall niche overlap for the ensemble D is calculated by using the model weights of each algorithm using the relative CBI as the weighting parameter between the three models. We use the asymmetric background similarity test from the *ENMtools* package in R to compare the overlap between two population niches of the same species. This test is used to infer whether two populations occupy significantly divergent ecological niches or if observed differences are due to background environmental variation. Low niche overlap values coupled with significant test results suggest true niche divergence, whereas non-significant results imply niche conservatism or shared environmental requirements.

Each of the 21 paired areas were given a 4-character identifier where the 2-digit codes of each ocean area are combined (e.g. North Atlantic and North Pacific – NA-NP; see Figure 1). To separate the effects of available environmental conditions on niche overlap, a null model is generated by constructing an ENM using the three algorithms used for the model ensembles. In each case the presence data from one ocean area with a subset of background samples from the other ocean basin, repeating the process 100 times to generate a null model distribution of D (D_null_) and *vice versa* to produce two D_null_. As with the true niche overlap D we use the CBI model weights of the three algorithms to generate an ensemble D_null_. To determine if the niches are conserved, neutral or diverged we compare D with the 95% confidence interval of both D_null_. If D is greater than the upper limit of the 95% CI of D_null_ then the true niche overlap is ***greater*** than what would be expecting by chance and the niche is ***conserved*** while if D is less than the lower limit of the 95% CI of D_null_ then the true niche overlap is ***less*** than what would be expected by chance and has ***diverged***. If D is within the 95% CI of D_null_ the niche is considered ***neutral***.

**Niche divergence assessed using principal component analysis**

The PCA method provides an additional level of confirmation of the niche differences, while the loadings of each environmental variable along each axes gives an indication of which gradients are driving any differences between species populations. For species with > 1000 observations in an ocean basin a subset of 1000 observations are selected to construct the PCA. We extract the environmental conditions from both ocean basins and select a subset of these such that the ratio of background observations: species observations are 10:1. An elbow plot was used to find the threshold for the number of axes that identifies most of the variation. The niche overlap is determined by calculating the difference in the mean scores for the presence locations between both ocean basins along each principal component axis. The null model for each principal component axis was determined by using 1000 jack-knife replicates to generate a 95% confidence interval of the differences in background observations. If the true niche overlap is ***less*** than the lower limit of the 95% CI of the null model, then the population niches are said to be conserved. If the true niche overlap is ***greater*** than the upper limit of the 95% CI of the null model, then the population niches are said to be diverged. If the niche overlap is within the 95% CI of the null model the niches are said to be neutral. Diverged niches are tested using a *t*-test with a Bonferroni correction for significance. This process is repeated for all species and area combinations to produce a dataset of species and areas with evidence of divergence.

**Testing for sampling bias and variation in sampling occurrences**

We test for the potential that differences in sampling occurrences could bias the niche overlap estimates between two populations. For example, observation of species in area A might contain only 50 observations while in area B there may be > 1000 present. We use a workflow that involves resampling the presence points for two separate populations being compared, for varying numbers of matched observations, selecting the same number of observations from each area, from 25 points up to 1000. We look at the changes in the ensemble niche overlap D for each species containing all observations against the resampled points for each of the different sample sizes estimated. The calculation of the resampled niche overlap is performed in the same way as described previously. Significant changes in D or random variation in D for increased number of observations would suggest that niche divergence could be driven by imbalances in the observation frequency in both areas. We also perform a simple logistic regression on the final classification of paired-area comparisons where the binary classification divergence or non-divergence is compared with the difference in the observation number between the two areas.

**Environmental relationships of niche divergence for copepod species**

Enough data were available for the copepod group to examine the potential environmental drivers of niche divergence between paired areas. Here we combined the dataset of niche divergence classification between paired areas with the mean niche values for each species obtained from the global ENM ensembles. The mean niche gives us an indication of the mean affinity of each species to environmental gradients averaged across all paired-areas. This gives us an indication of what habitat each species are likely to occupy and what habitats are more or less likely to encounter niche divergence. We used hierarchical generalised additive models (HGAM) to test whether the likelihood of niche divergence between ocean basins is affected by differences in the global niche for each species. HGAM differ from traditional GAM as they allow for the relationships between predictor and response variables to vary across different groups (Pedersen et al., 2019). HGAM were developed for the presence or absence of niche divergence between paired areas as the binomial dependant variable. Model selection was performed using the selection penalties approach as recommended by Pedersen et al. (2019) to determine the final choice of smoothers in the final model. Essentially, it allows for the shrinkage of different smoothers to zero effective degrees of freedom, removing these from the model. All variables were first considered as a global smoother. Significant smoothers were then investigated for group-level effects. With limited data between paired-areas, estimates of both fixed and random effects may become biased and as a result we excluded paired areas with <10 comparisons for copepods resulting in 14 of the 21 areas retained and 552 paired area comparisons. After selection the final model was of the form:

Div ~ s(Bathymetry, k=4, bs="tp")+s(Temperature, kn=4, m=2, bs="tp")+

s(Temperature, by=Paired Areas, kn=4, m=1, bs="fs")+s(Paired Areas, bs="re", k=12)

The model fits a global level trend for the bathymetry and temperature. Temperature also allows for different group-level trends with different smoothness between different paired areas with the maximum smoothness or knots set to 4 to prevent overfitting. Paired areas will show significance with temperature if the group level trend is significantly different than the global pattern. We also included the paired areas as a random effect to account for the variation of species between paired areas.

**References (Not present in main text)**

Schroeder, P.J. and Jenkins, D.G., 2018. How robust are popular beta diversity indices to sampling error?. *Ecosphere*, *9*(2), p.e02100.
